# Dendritic excitation–inhibition balance shapes cerebellar output during motor behaviour

**DOI:** 10.1038/ncomms13722

**Published:** 2016-12-15

**Authors:** Marta Jelitai, Paolo Puggioni, Taro Ishikawa, Arianna Rinaldi, Ian Duguid

**Affiliations:** 1 Centre for Integrative Physiology and Patrick Wild Centre, University of Edinburgh, Edinburgh Medical School: Biomedical Sciences, Hugh Robson Building, George Square, Edinburgh EH8 9XD, UK; 2 Institute of Experimental Medicine, Hungarian Academy of Sciences, H-1083 Budapest, Hungary; 3 Institute for Adaptive and Neural Computation, School of Informatics, University of Edinburgh, Edinburgh EH8 9AB, UK; 4 Neuroinformatics Doctoral Training Centre, School of Informatics, University of Edinburgh, Edinburgh EH8 9AB, UK; 5 Department of Pharmacology, Jikei University School of Medicine, Tokyo 105-8461, Japan

## Abstract

Feedforward excitatory and inhibitory circuits regulate cerebellar output, but how these circuits interact to shape the somatodendritic excitability of Purkinje cells during motor behaviour remains unresolved. Here we perform dendritic and somatic patch-clamp recordings *in vivo* combined with optogenetic silencing of interneurons to investigate how dendritic excitation and inhibition generates bidirectional (that is, increased or decreased) Purkinje cell output during self-paced locomotion. We find that granule cells generate a sustained depolarization of Purkinje cell dendrites during movement, which is counterbalanced by variable levels of feedforward inhibition from local interneurons. Subtle differences in the dendritic excitation–inhibition balance generate robust, bidirectional changes in simple spike (SSp) output. Disrupting this balance by selectively silencing molecular layer interneurons results in unidirectional firing rate changes, increased SSp regularity and disrupted locomotor behaviour. Our findings provide a mechanistic understanding of how feedforward excitatory and inhibitory circuits shape Purkinje cell output during motor behaviour.

## Abstract (web summary)

Here, the authors combine *in vivo* patch-clamp recordings and optogenetics to show that balanced dendritic excitation and inhibition provides a sensitive ‘push-pull' mechanism that generates the bidirectional modulation of Purkinje cell SSp output necessary for normal locomotor behaviour.

Cerebellar Purkinje cells (PCs) encode sensorimotor information during locomotion via bidirectional modulation (that is, increase or decrease) of their simple spike (SSp) firing rates^1^,^2^,^3^,^4^,^5^. In the intact cerebellum, PCs display baseline firing rates of ∼50 Hz, which can be enhanced (up to a maximum firing frequency of 250 Hz) or suppressed (to ∼5 Hz) during movement. This bidirectional modulation is thought to be dependent upon the interplay between somatic and dendritic intrinsic conductances^6^,^7^,^8^,^9^ and activity-dependent changes in the balance between excitation and inhibition^10^,^11^,^12^,^13^. PCs—the axons of which constitute the sole output from the cerebellar cortex—receive strong feedforward inhibition (FFI) from molecular layer interneurons (MLIs)^11^,^14^,^15^,^16^,^17^. This fast, direct inhibition opposes the effects of excitatory input from cerebellar granule cells (GCs) to modulate the rate and temporal dynamics of PC SSp output^15^,^18^. While previous experimental and modelling studies have highlighted the importance of excitatory and inhibitory synaptic input in regulating PC SSp dynamics and motor control^10^,^11^,^12^,^15^,^19^,^20^,^21^, how these inputs combine to generate bidirectional PC SSp output during motor behaviour remains unresolved.

To elucidate the mechanisms underpinning SSp modulation, we aimed to test three biologically plausible synaptic input models *in vivo* (Fig. 1a,b). Model 1 describes bidirectional changes (that is, an enhancement or suppression) in feedforward excitation (FFE) from GCs accompanied by a concomitant increase in FFI from MLIs. In this scenario, if the steady-state level of inhibitory input is outweighed by an increase in excitation, SSp firing rates increase, whereas if the level of excitatory input falls during locomotion, inhibition dominates to reduce the SSp firing rate. Although GCs appear to display unidirectional firing rate changes during locomotion^22^, altered sensory input to preferred versus non-preferred stimuli has the potential to drive bidirectional firing rate modulation in GCs^23^. Model 2 describes bidirectional inhibitory input modulation with a concomitant increase in excitation, where reduced inhibition allows FFE to dominate increasing SSp firing rates, whereas enhanced inhibition has the opposing effect. Mutual inhibition of MLIs has the potential to produce complex patterns of enhanced and suppressed FFI to PCs^15^,^24^. Finally, model 3 describes the unidirectional but variable enhancement of FFE and FFI, the ratio of which dictates the magnitude and direction of PC SSp firing rate changes.

To test these synaptic input models *in vivo*, we combined dendritic and somatic patch-clamp recordings from PCs, optogenetic silencing of MLIs and quantitative behavioural analysis. We show that GCs generate a sustained depolarization of PC dendrites during movement, which is counterbalanced by variable levels of FFI from MLIs. Locomotion-dependent modulation of the balance between excitation and inhibition generates depolarizing or hyperpolarizing dendritic membrane potential (*V*_m_) changes that linearly transform into bidirectional modulation of PC SSp output. Disrupting the excitation–inhibition balance by selectively silencing MLIs abolishes bidirectional firing rate changes, increases the rate and regularity of SSps, and disrupts normal locomotor function. Together, our findings provide a mechanistic understanding of how feedforward excitatory and inhibitory circuits regulate the somatodendritic excitability of PCs during self-paced motor behaviour.

## Results

### Modulation of PC SSp output during locomotion

**Figure 1:**
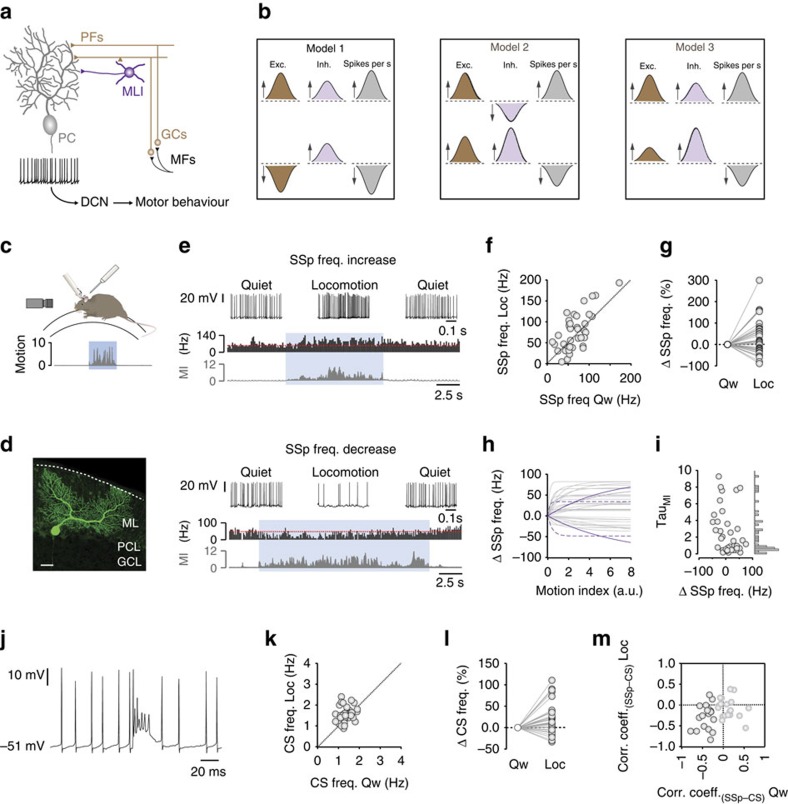
Bidirectional Purkinje cell SSp modulation during locomotion. (**a**) Schematic showing feedforward circuitry of the cerebellum. DCN, deep cerebellar nuclei; GCs, granule cells; PC, Purkinje cell; PFs, parallel fibres; MFs, mossy fibres. (**b**) Feedforward input models underpinning locomotion-dependent bidirectional PC simple spike (SSp) modulation. exc, excitation; freq., frequency; inh, inhibition. (**c**) Recording configuration in awake mice where locomotion was captured using digital video. (**d**) Intracellular biocytin labelling of a PC via the recording electrode. Scale bar, 30 μm. GCL, granule cell layer; PCL, Purkinje cell layer; ML, molecular layer. (**e**) Somatic voltage recordings, spike rate histograms and associated motion index values (MI; dark grey) from two PCs during quiet wakefulness and voluntary locomotion (blue). Dashed red line denotes average firing rate during quiet wakefulness. (**f**) Purkinje cell quiet wakefulness SSp firing rate (Qw) as a function of locomotion-related SSp firing rate (Loc, *n*=38 cells, *N*=33 mice). Symbols represent individual PCs and dotted line represents unity. (**g**) Average change in PC SSp firing rate during quiet wakefulness (Qw) and locomotion (Loc; *n*=38 cells, *N*=33 mice). (**h**) Average change in PC SSp firing rate as a function of increasing movement. Grey lines represent exponential fits to the data in individual cells, solid purple lines represent PCs that increase/decrease their firing rates relative to the magnitude of movement and dashed purple lines represent PCs that are highly sensitive only to small movements (*n*=38 cells, *N*=33 mice). a.u., arbitrary units. (**i**) Distribution of Tau_MI_ values taken from the exponential fits shown in **h**. (**j**) Representative example of a climbing fibre-mediated complex spike (CS). (**k**) Purkinje cell quiet wakefulness (Qw) CS firing rate as a function of locomotion-related CS firing rate (*n*=38 cells, *N*=33 mice). Symbols represent individual PCs and dotted line represents unity. (**l**) Average change in PC CS firing rate during quiet wakefulness (Qw) and locomotion (Loc; *n*=38 cells, *N*=33 mice). (**m**) Distribution of CS versus SSp firing rate correlation coefficients during quiet wakefulness (Qw) and locomotion (Loc). Black circles denote PCs that displayed reciprocal firing rates during quiet wakefulness or locomotion (*n*=18/38 cells, *N*=33 mice).

To investigate the cellular mechanisms underpinning bidirectional PC SSp modulation during locomotion^2^,^3^,^4^,^5^,^25^, we made somatic patch-clamp recordings from adult mouse PCs along the apex of lobule V during self-paced, voluntary locomotion on a single axis, cylindrical treadmill (Fig. 1c). The relationship between PC SSp firing rates and changing behavioural state (that is, quiet wakefulness versus self-paced locomotion) was measured by extracting a motion index (MI) from digital video sequences and aligning this to our electrophysiological recordings (Fig. 1c–e; see Methods)^26^. Although the MI does not provide information regarding specific aspects of limb movement/coordination, it does provide a sensitive measure of whole-body movement including movement preparation, which precedes rotation of the treadmill (Supplementary Fig. 1).

PCs were identified based on their distinct electrophysiological signatures—including the occurrence of complex spikes (CSs) at ∼1 Hz (ref. 27)—and morphology (Fig. 1d,e,j; Supplementary Tables 1 and 2). During quiet wakefulness, PCs displayed unimodal subthreshold *V*_m_ distributions (23/24 cells) centred around −50 mV (−50.5±0.47 mV, range −45.8 to −54.1 mV; Supplementary Fig. 2B), and a wide range of somatic SSp firing rates (mean 66.9±4.9 Hz, range 12.4–171.9 Hz, *n*=38 cells from *N*=33 mice). During locomotion, PC SSp firing rates increased, decreased or remained unaffected (*n*=21/38, 14/38, and 3/38 cells, respectively, based on the correlation coefficients between firing rate and MI) effectively forming a continuum of positive to negative SSp firing rate changes (Fig. 1e–g; Supplementary Fig. 2C). The direction of SSp modulation during locomotion did not depend on the quiet wakefulness firing rate of individual PCs (*r*=−0.02, *P*=0.91, *n*=38 from *N*=33 mice; Supplementary Fig. 2D,E)—that is, cells that displayed high firing rates during quiet wakefulness did not show a bias towards decreasing their firing rates during locomotion or vice versa^5^. Moreover, the direction of each SSp firing rate change was maintained across successive bouts of locomotion, suggesting individual PCs reliably encode similar locomotion-based movements with comparable changes in SSp firing rate (Supplementary Fig. 3). PCs also differed in their sensitivity to movement, with some cells displaying SSp firing rate changes that correlated with the magnitude of movement (MI=0–8, Tau_MI_ ∼1–10, solid purple line; Fig. 1h,i), while other PCs were highly sensitive to small movements associated with movement preparation or initiation (MI=0–2, Tau_MI_ ∼0–1), but insensitive to increased movement during locomotion (asymptotic; MI=2–8; dashed purple line; Fig. 1h,i; Supplementary Fig. 4).

During quiet wakefulness, PCs also displayed climbing fibre-mediated CS activity at ∼1.4 Hz (1.36±0.05 range 0.86–1.9, *n*=38; Supplementary Table 2), where approximately half of the cells exhibited reciprocal CS–SSp firing rates (*r*=−0.41±0.04, *n*=18/38, *P*<0.05). During locomotion, CS activity was also enhanced, suppressed or unaffected, forming a continuum of positive to negative rate changes and, in general, the SSp-CS reciprocity was maintained (*r*=−0.54±0.05, *n*=10/38, *P*<0.05; Fig. 1j–m).

### Dendritic membrane potential changes during locomotion

**Figure 2:**
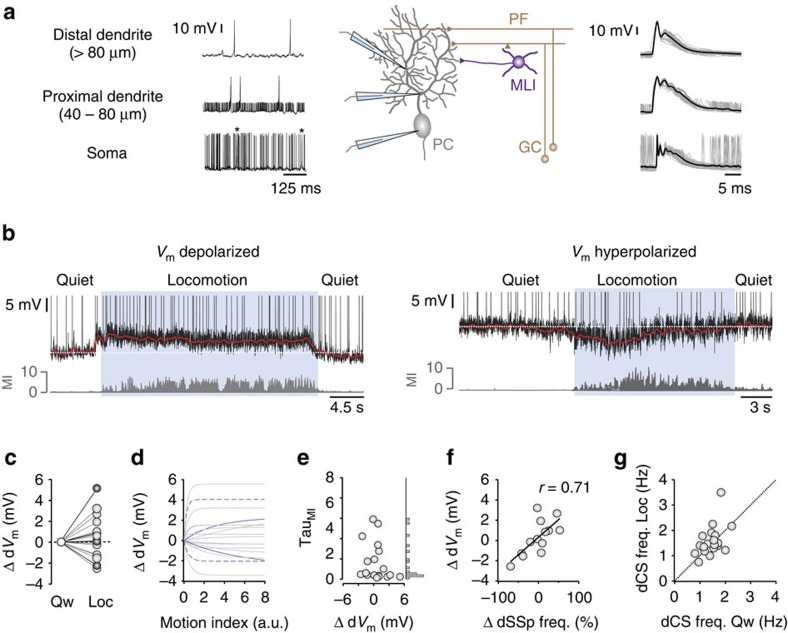
Purkinje cell d*V*_m_ changes during locomotion. (**a**) Schematic showing PC recording configurations (GC, granule cell; MLI, molecular layer interneuron; PC, Purkinje cell; PF, parallel fibre) and representative somatic (lower trace), proximal dendritic (middle trace, ∼60 μm from soma) and distal dendritic (upper trace, ∼100 μm from soma) voltage recordings from three PCs *in vivo*. Asterisks denote complex spikes. Right—dendritic calcium spike waveforms (50 consecutive traces overlaid (grey) and average *V*_m_ (black)). (**b**) Representative dendritic voltage recordings from two different PCs that depolarize (upper trace) or hyperpolarize (lower trace) during self-paced locomotion (blue shading). Motion index (MI, grey) defines the magnitude and duration of each locomotion bout. Note dendritic calcium spikes have been truncated to improve visualization of dendritic *V*_m_ (d*V*_m_) changes. Red lines depict smoothed average *V*_m_. (**c**) Average PC d*V*_m_ changes (Δd*V*_m_) during self-paced locomotion (*n*=19 cells, *N*=17 mice). Black filled circles depict d*V*_m_ changes in distal dendritic recordings (*n*=6 cells) and grey symbols depict d*V*_m_ changes in proximal dendritic recordings (*n*=13 cells). (**d**) Average change in d*V*_m_ (Δd*V*_m_) as a function of increasing movement (MI). Grey lines represent exponential fits to the data in individual cells, solid purple lines represent examples of d*V*_m_ changes that are relative to the magnitude of movement and dashed purple lines represent cells that are highly sensitive to small movements associated with movement preparation or initiation (*n*=19 cells, *N*=17 mice). a.u., arbitrary units. (**e**) Distribution of Tau_MI_ values taken from the exponential fits shown in **d**. (**f**) Relationship between Δd*V*_m_ and percentage change in dSSp firing rate during self-paced, locomotion (*n*=13 cells, *N*=11 mice). Filled symbols represent the data from individual PCs and the solid line is a linear fit to the data *r*=0.71, *P*<0.01. (**g**) Average dendritic calcium spike frequency during quiet wakefulness and locomotion (*n*=19 cells, *N*=17 mice). Filled circles represent individual cells and dotted line represents unity.

To investigate the extent to which movement-related changes in dendritic *V*_m_ shapes PC spike output, we made dendritic patch-clamp recordings from PCs during self-paced locomotion. The somatodendritic structure of PCs, with primary and secondary dendrites measuring ∼3–8 μm in diameter, make them amenable to intracellular recording *in vivo* (Fig. 2a). While somatic recordings were identified by the presence of full-height simple (SSps) and CSs, dendritic recordings were identified by the presence of large overshooting calcium spikes and smaller ‘spikelets' (Fig. 2a; Supplementary Table 2). Since PC dendrites do not support active backpropagation of action potentials^6^,^21^,^28^, dendritic ‘spikelets' reflect passive backpropagation of SSps from the soma to the dendrite (dendritic SSps (dSSps); 3–10 mV in amplitude ∼40–80 μm from soma, <3 mV in amplitude >80 μm from soma, *n*=19 from *N*=17 mice; Fig. 2a,b).

During locomotion, we observed a continuum of dendritic *V*_m_ (d*V*_m_) changes ranging from a moderate hyperpolarization (−2.6 mV) to a modest depolarization (5.2 mV; *n*=19 cells; Fig. 2b,c), which persisted for the duration of each movement bout. Although quiet wakefulness d*V*_m_ distributions were relatively broad (d*V*_m_ s.d. 2.26±0.11 mV, *n*=19 cells) locomotion induced a clear leftward (that is, hyperpolarization) or rightward (that is, depolarization) shift in d*V*_m_ (Supplementary Fig. 5). Similar to somatic SSp firing rates, PC d*V*_m_ displayed differing sensitivity to movement, with some cells displaying d*V*_m_ changes that correlated with the magnitude of movement (MI=0–8, Tau_MI_ ∼1–8, solid purple line), while other PCs were highly sensitive to small movements associated with movement preparation or initiation (MI=0–2, Tau_MI_ ∼0–1), but insensitive to increased movement during locomotion (asymptotic; MI=2–8; dashed purple line, Fig. 2d,e).

By exploiting our ability to simultaneously measure d*V*_m_ and dSSp changes in proximal dendritic recordings during locomotion (Fig. 2a), we found that movement-related d*V*_m_ changes strongly correlated with the magnitude and direction (that is, increase versus decrease) of SSp firing rate changes (*n*=13 cells from *N*=11 mice, *r*=0.71, *P*=0.007), where cells that displayed a dendritic hyperpolarization were associated with a reduction in dSSp firing rate and vice versa (Fig. 2f). Changes in dendritic calcium spike activity mirrored changes in the CS activity measured at the soma (compare Figs 1k and 2g). Together, our results show that relatively small changes in PC d*V*_m_ (0–5 mV) linearly transform into robust bidirectional changes in somatic SSp firing rates during locomotion, and that intracellular dendritic and somatic recordings *in vivo* provide a powerful method to investigate how FFE and FFI shape PC output during motor behaviour.

### Enhanced GC activity during locomotion

**Figure 3:**
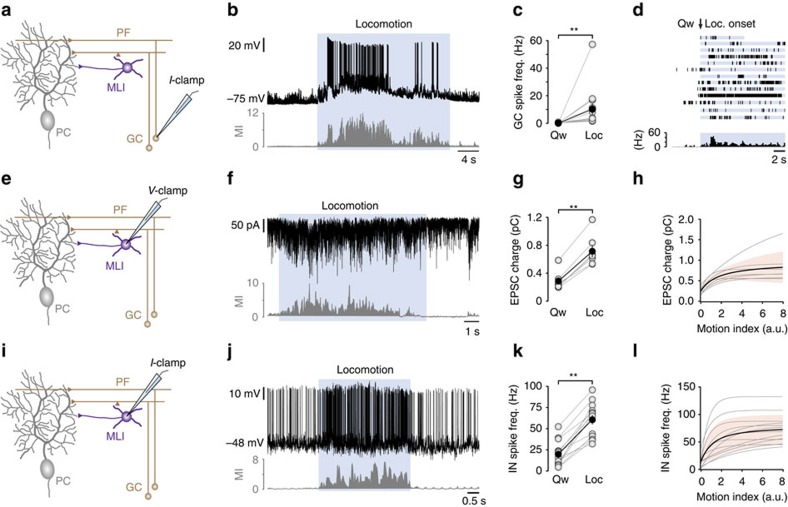
Recruitment of feedforward excitatory and inhibitory circuits during locomotion. (**a**) Schematic showing GC current (*I*)-clamp recording configuration. GC, granule cell; MLI, molecular layer interneuron; PC, Purkinje cell; PF, parallel fibre. (**b**) Representative voltage trace recorded from a GC during quiet wakefulness and locomotion (blue shading). (**c**) Average change in firing rate during quiet wakefulness (Qw) and locomotion (Loc). Grey symbols represent the data from individual GCs and black symbols represent mean±s.e.m., **P<0.001 Wilcoxon matched-pairs signed-rank test (*n*=13 cells, *N*=13 mice). (**d**) Raster plot (upper panels) and average firing rate histogram (lower panel, bin size=200 ms) of a population of GCs during quiet wakefulness (Qw) and locomotion (Loc; blue shading; *n*=13 cells, *N*=13 mice). (**e**) Schematic showing MLI voltage (*V*)-clamp recording configuration. (**f**) Representative current trace recorded at −70 mV (upper panel) from a MLI during quiet wakefulness and locomotion (blue shading). (**g**) Average EPSC charge transfer recorded during quiet wakefulness (Qw) and locomotion (Loc). Grey symbols represent data from individual MLIs, black symbols represent mean±s.e.m., **P<0.01 two-tailed *t*-test (*n*=7 cells, *N*=6 mice). (**h**) Average charge transfer as a function of increasing movement (motion index). Thin grey lines represent exponential fits to the data in individual cells, black line represents the average and pink shading the s.d. of the mean (*n*=7 cells, *N*=6 mice). (**i**) Schematic showing MLI *I*-clamp recording configuration. a.u., arbitrary units. (**j**) Representative voltage trace recorded from a MLI during quiet wakefulness and locomotion (blue shading). (**k**) Average firing rate of MLIs during quiet wakefulness (Qw) and locomotion (Loc; *n*=13 cells, *N*=13 mice). Grey symbols and connecting lines represent the data from individual MLIs, and black symbols represent mean±s.e.m. **P<0.01 two-tailed *t*-tests. (**l**) Average change in MLI firing rate as a function of increasing movement (motion index). Thin grey lines represent exponential fits to the data in individual cells, black line represents the average across all cells and pink shading the s.d. of the mean (*n*=13 cells, *N*=13 mice). a.u., arbitrary units.

To investigate the extent to which feedforward excitatory circuits are engaged during self-paced locomotion, we recorded from individual GCs along the apex of lobule V (Fig. 3a). GCs were identified based on their electrophysiological properties^22^,^23^,^29^,^30^,^31^ and depth from the pial surface (>350 μm; Supplementary Table 3). At rest, GCs displayed low baseline firing rates (0.14±0.08 Hz, *n*=13 cells from *N*=13 mice), which markedly increased after locomotion onset (10.22±4.22 Hz, *n*=13, *P*<0.0001; Fig. 3b,c), consistent with previous findings^22^. The frequency and pattern of movement-evoked spiking in individual GCs were highly variable, with some GCs displaying continuous high-frequency firing, while others displayed intermittent spike bursts (Fig. 3d; Supplementary Table 4). The population-based response of GCs appeared as a gradual increase in spike rate during locomotion onset, rising to a peak around 2 s—potentially reflecting the relatively slow depolarization to threshold in some GCs—followed by sustained firing for the duration of the movement bout. The consistent increase in GC firing rates (see also ref. 22) suggests that GC activity alone cannot drive bidirectional d*V*_m_ or SSp firing rate changes in PCs during locomotion (Fig. 1b). Although we undersampled the GC population, our results are consistent with GCs providing strong, sustained feedforward excitatory input to downstream PCs and MLIs during self-paced locomotion.

### GCs drive interneuron firing during locomotion

To assess the recruitment of feedforward inhibitory circuits by GCs, we made voltage- and current-clamp recordings from MLIs during quiet wakefulness and voluntary locomotion (Fig. 3e–j). As with GCs, MLIs were identified based on their electrophysiological properties and depth from the pial surface (range 126–296 μm; Supplementary Table 5)^30^,^32^. During quiet wakefulness, interneurons (INs) received high-frequency barrages of excitatory input where the mean amplitude and frequency of parallel fibre inputs were 52.7±10.2 pA and 191.5±23.9 Hz, respectively, with an average 20–80% rise time of 0.28±0.02 ms (*n*=7 neurons from *N*=6 mice; Fig. 3f; Supplementary Fig. 6). Given the low rate of GC activity *in vivo* (Fig. 3b–d)^22^,^29^,^30^,^31^, INs likely receive high rates of spontaneous action potential-independent input or low frequency input from a large population of GCs even during periods of rest. Although we did not test the contribution of climbing fibres directly^32^,^33^, it is possible that climbing fibres contribute to the basal excitatory postsynaptic current (EPSC) rate and recruitment of FFI^34^,^35^. During periods of locomotion, the phasic charge transfer of EPSCs—a compound measure of EPSC amplitude and frequency (Methods)—increased in all cells (Qw=0.29±0.05, Loc=0.72±0.09, *P*<1.0 × 10^−3^, *n*=7 neurons from *N*=6 mice; Fig. 3g) and was highly sensitive to changes in the magnitude of movement (Pearson correlation coefficient 0.51±0.03, *n*=7), where locomotion onset triggered a sustained increase in charge that co-varied with changes in MI (Fig. 3h; Supplementary Figs 4 and 6).

We next investigated how increased parallel fibre input transformed into behaviourally relevant output spike patterns in MLIs. We found that all MLIs increased their firing rates during movement, rising from an average of 20 Hz during quiet wakefulness to ∼60 Hz during locomotion (quiet wakefulness 19.8±4.2 Hz, locomotion 60.5±5.7, *n*=13 neurons from *N*=13 mice, *P*=1 × 10^−4^; Fig. 3j,k). Similar to changes in EPSC charge transfer, MLI firing rates were highly sensitive to changes in movement (Pearson's correlation coefficient 0.71±0.04, *n*=13), with locomotion triggering a sustained increase in firing that co-varied with changes in MI (Fig. 3j–l; Supplementary Figs 4 and 7). The distribution of charge/firing rate versus MI Tau values in MLI (Fig. 3h,l; Supplementary Figs 6 and 7) were similar to those observed for firing rate changes versus MI in PCs (Fig. 1), suggesting that GC activity likely drives the differential sensitivity to movement in both types of molecular layer neurons. Importantly, peak and steady-state movement-related firing rates of MLIs were highly variable across individual MLIs, generating variable, but sustained, FFI to downstream PCs.

To explore the relationship between GC input and MLI spike output, we generated an input–output curve by plotting the average charge transfer versus firing rate for each binned MI value (200 ms bin size; that is, combining input and output data from different populations of cells shown in Fig. 3h,l; Methods). Consistent with *in vitro* and modelling predictions, we found that MLIs encode locomotion-dependent changes in GC input with linear changes in firing rate, with input and output rates being highly variable across MLIs (EPSCs 0.53–1.17 pC, *n*=7; spikes 32.0–95.8 Hz, *n*=13) (Fig. 3e–l; Supplementary Fig. 7F).

### Excitation–inhibition balance regulates PC d*V*_m_

**Figure 4:**
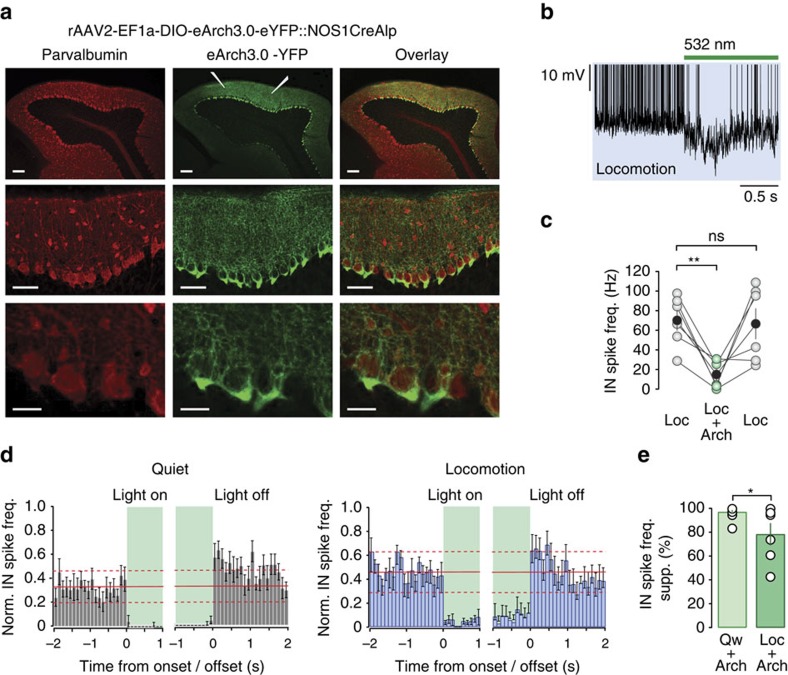
Arch 3.0-mediated silencing of molecular layer interneurons. (**a**) Low- and higher-magnification confocal images of parvalbumin (PV, red) and eArch3.0-eYFP labelling (green) along the apex of lobule V of the cerebellar vermis. Parasagittal sections of lobule V (upper panel, scale bar, 100 μm) were cut 9 days post virus injection. Two injections (white electrodes, middle panel) were performed at anterior and posterior locations in the craniotomy to ensure maximal viral infection across lobule V. Lower panels—higher-magnification images of lobule V highlighting the cell-selective expression of Arch 3.0 in MLIs (middle panels, scale bar, 50 μm; bottom panels, scale bar, 20 μm). (**b**) Example of light-evoked silencing of a MLI (green bar, 2 s pulse of 532 nm light) during locomotion (blue shading). Note spikes have been truncated to improve visualization of the photo-induced hyperpolarization. (**c**) Average firing rate of MLIs during locomotion (Loc), locomotion plus light activation (Loc+Arch) and after cessation of the light stimulus (Loc). Grey and green symbols and connecting lines represent the data from individual MLIs, and black symbols represent mean±s.e.m.**P<0.01, ns, not significant, two-tailed *t*-tests (*n*=6 cells, *N*=6 mice). (**d**) Normalized MLI firing frequency histogram (bin size=100 ms) aligned to the onset and offset of 532 nm light stimulation (green shading) during quiet wakefulness (grey left hand panels, *n*=9 cells, *N*=8 mice) and locomotion (blue right hand panels, *n*=6 cells, *N*=6 mice). Solid red line depicts the mean frequency before light stimulation and dashed lines indicate 2 × s.d. of the mean. (**e**) Mean percentage suppression of MLI firing frequency after Arch 3.0 stimulation during quiet wakefulness (Qw+Arch, *n*=9, *N*=8 mice) and locomotion (Loc+Arch, *n*=6, *N*=6 mice). Open circles represent the data from individual MLIs and bars represent mean±s.e.m., *P<0.05, two-tailed *t*-test. freq., frequency; Norm., normalization; supp., suppression.

To directly investigate the balance between FFE and FFI in PCs, we combined cell-selective optogenetic silencing of MLIs with intracellular dendritic recordings from PCs. Archaerhodopsin 3.0 (Arch 3.0), a light-activated proton pump^36^, was targeted to MLIs using a *Nos1Cre* transgenic mouse line^37^,^38^ and viral-mediated gene transfer (rAAV2-EF1a-DIO-eArch3.0-eYFP::*Nos1Cre*; Fig. 4a; Supplementary Fig. 8). To assess the efficiency of Arch 3.0, we performed whole-cell and cell-attached recordings from MLIs and found that light activation (2–4 s pulses of 532 nm light) hyperpolarized the mean *V*_m_ (quiet wakefulness Δ*V*_m_ −15.4±3.2 mV, *n*=6 from *N*=6 mice; locomotion Δ*V*_m_ −4.3±2.3 mV, *n*=3 from *N*=3 mice), significantly reducing MLI firing rates during locomotion (Loc: 70.1±10.5 Hz, Loc+Arch: 14.8±5.6 Hz, *n*=6 neurons from *N*=6 mice, *P*=4.6 × 10^−3^; Fig. 4b–e). The onset and offset latencies of Arch 3.0-mediated effects in MLIs were 17.0±8.3 and 14.8±10.6 ms, respectively. Although silencing MLIs during quiet wakefulness induced a small but consistent rebound firing after stimulus cessation (<300 ms), no significant rebound activity was observed after Arch 3.0 activation during locomotion (Fig. 4d). Moreover, light activation in the absence of Arch 3.0 expression did not affect the firing frequency of MLIs (Supplementary Fig. 8).

**Figure 5:**
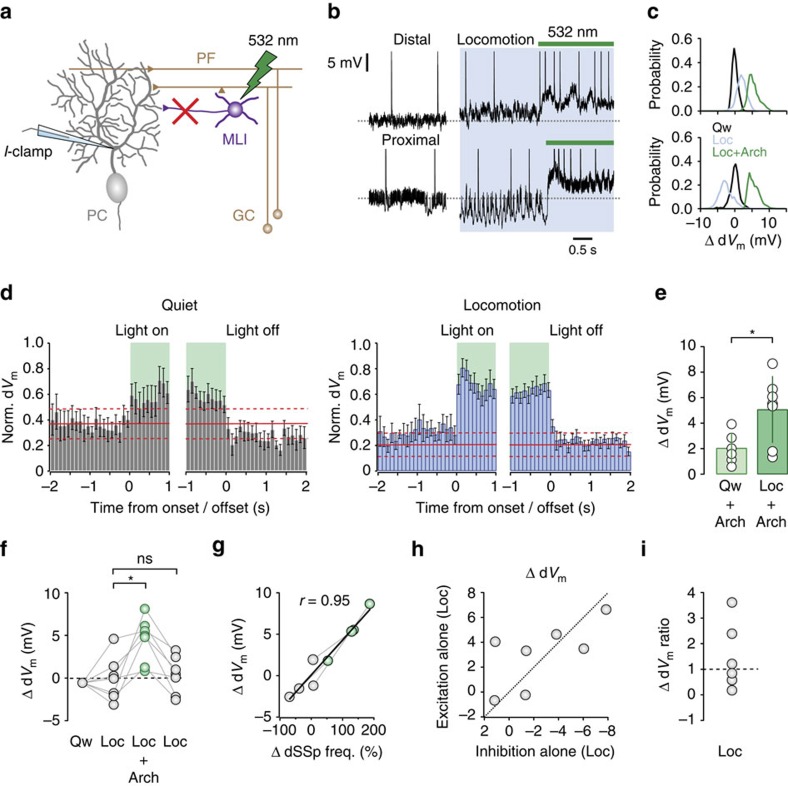
Excitation–inhibition balance regulates Purkinje cell d*V*_m_. (**a**) Schematic showing MLI current (*I*)-clamp recording configuration during light activation of Arch 3.0 (green, 532 nm). (**b**) d*V*_m_ recordings from two PCs during quiet wakefulness, locomotion (blue) and locomotion during light stimulation (green). (**c**) Normalized d*V*_m_ distributions (from **b**) during quiet wakefulness (black), locomotion (blue) and locomotion plus light stimulation (green). (**d**) Normalized PC d*V*_m_ (bin size=100 ms) aligned to the onset and offset of light stimulation (green) during quiet wakefulness (grey, *n*=6 cells, *N*=6 mice) and locomotion (blue, *n*=7 cells, *N*=6 mice). Red line depicts mean d*V*_m_ during quiet wakefulness±2 × s.d. (**e**) Average Δd*V*_m_ after light stimulation during quiet wakefulness (Qw+Arch, *n*=6, *N*=6 mice) and locomotion (Loc+Arch, *n*=7, *N*=6 mice). Circles represent the data from individual MLIs and bars represent mean±s.e.m., *P<0.05, two-tailed *t-*test. (**f**) Average locomotion-related changes in d*V*_m_ (Δd*V*_m_) before (grey, Loc), during (green, Loc+Arch) and after (grey, Loc) light stimulation. Connecting lines represent the data from individual PCs *P<0.05, ns, not significant, two-tailed *t*-tests (*n*=7 cells, *N*=6 mice). (**g**) Relationship between Δd*V*_m_ and ΔdSSp frequency during locomotion in the presence (grey) and absence (green, *n*=4, *N*=3 mice) of feedforward inhibition. Grey connecting lines represent the data from individual PCs and thick line is a linear fit to the data (*r*=0.95, *P*<0.01). (**h**) Estimated dendritic excitation/inhibition balance. Inhibitory effect on d*V*_m_ was calculated using Δd*V*_m_ Loc−(Δd*V*_m_ Loc+Arch)+(Δd*V*_m_ Qw+Arch) (*n*=7, *N*=6 mice), effects of excitation alone were calculated using (Δd*V*_m_ Loc+Arch)−(Δd*V*_m_ Qw+Arch). Dotted line represents unity. (**i**) Estimated ratio of effects of excitation ((Δd*V*_m_ Loc+Arch)−(Δd*V*_m_ Qw+Arch)) versus inhibition (Δd*V*_m_ Loc−(Δd*V*_m_ Loc+Arch)+(Δd*V*_m_ Qw+Arch)) on Δd*V*_m_ during locomotion. Ratio was calculated using absolute values of Δd*V*_m_ change (*n*=7, *N*=6 mice). Norm., normalization.

Blocking FFI revealed a strong depolarization and rightward shift in PC dendritic *V*_m_ distributions—irrespective of whether Δd*V*_m_ was depolarizing or hyperpolarizing in the absence of light stimulation—and increased dSSp firing rates in all cells (*n*=7 from *N*=6 mice; Fig. 5a–c). The Arch 3.0-mediated effects on d*V*_m_ were only observed during light stimulation and returned to baseline levels after stimulus cessation (Fig. 5d). These results confirm that GCs provide unidirectional, sustained FFE to PCs that alone cannot account for the bidirectional firing rate changes observed during locomotion (Figs 1 and 5f,g). To estimate the Δd*V*_m_ ratio (excitation/inhibition) in individual PC dendrites (see Methods), we took advantage of the fact that PCs linearly transform excitatory and inhibitory inputs to perform a simple subtraction analysis^24^,^39^ taking into account the baseline excitatory and inhibitory input to PCs during quiet wakefulness (see Methods; Fig. 5d,e). We found that during locomotion, PCs received variable levels of inhibitory input that counteracted the effects of FFE to generate a range of Δd*V*_m_ ratios centred around unity (excitation>inhibition=Δd*V*_m_ ratio>1, excitation<inhibition=Δd*V*_m_ ratio<1; Fig. 5h,i). These results suggest that the fine balance between dendritic excitatory and inhibitory input provides a robust cellular mechanism to generate bidirectional SSp firing rate modulation during locomotion.

### Excitation–inhibition ratio regulates cerebellar output

**Figure 6:**
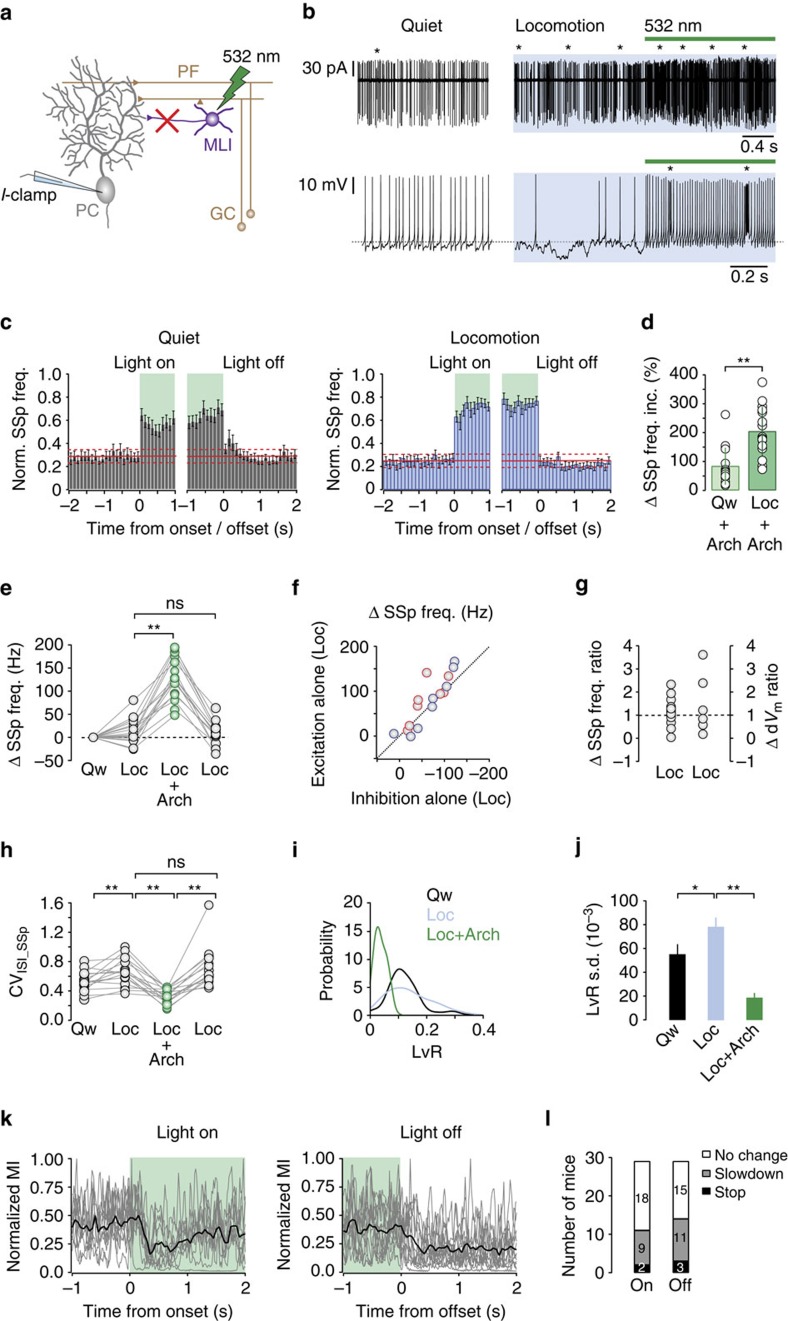
Excitation–inhibition balance shapes Purkinje cell SSp output during locomotion. (**a**) Schematic showing PC somatic recording configuration during light activation. (**b**) Cell-attached (upper) and whole-cell (lower) recordings from two PCs during quiet wakefulness, locomotion (blue) and locomotion plus light stimulation (green). Asterisks denote complex spikes. (**c**) Normalized PC SSp frequency histogram (bin size=100 ms) aligned to the onset and offset of 532 nm light stimulation (green) during quiet wakefulness (grey, *n*=16 cells, *N*=14 mice) and locomotion (blue, *n*=16 cells, *N*=14 mice). Solid red line depicts mean frequency during quiet wakefulness±2 × s.d. (**d**) Average change in PC SSp frequency after Arch 3.0 stimulation during quiet wakefulness (Qw+Arch) and locomotion (Loc+Arch). Circles represent the data from individual PCs and bars represent mean±s.e.m., **P<0.01, two-tailed *t*-test, (*n*=16 cells, *N*=14 mice). (**e**) Average ΔSSp frequency before (grey, Loc), during (green, Loc+Arch) and after (grey, Loc) light stimulation. Connecting lines represent the data from individual PCs, **P<0.01, ns, not significant, two-tailed *t*-tests (*n*=16 cells, *N*=14 mice). (**f**) Estimated effects of excitation and inhibition on PC SSp output. Inhibitory effect on SSp firing rate was calculated using ΔSSp Loc−(ΔSSp Loc+Arch)+(ΔSSp Qw+Arch) (*n*=16, *N*=14 mice), while the effects of excitation alone was calculated using (ΔSSp Loc+Arch)−(ΔSSp Qw+Arch). Red and blue circles represent PCs with high (>65 Hz) and low (<65 Hz) quiet wakefulness firing rates, respectively. Dotted line represents unity. (**g**) Ratio of effects of excitation ((ΔSSp Loc+Arch)−(ΔSSp Qw+Arch)) versus inhibition (ΔSSp Loc−(ΔSSp Loc+Arch)+(ΔSSp Qw+Arch)) on ΔSSp firing rate during locomotion. Ratio was calculated using absolute values of ΔSSp change and Δd*V*_m_ ratios were taken from Fig. 5 for comparison. (**h**) Average change in the CV of PC SSp inter-event intervals (CV_ISIs_SSp_) before (grey, Loc), during (green, Loc+Arch) and after (grey, Loc) light stimulation. Connecting lines represent data from individual PCs **P<0.01, ns, not significant, two-tailed *t*-tests (*n*=16 cells, *N*=14 mice). (**i**,**j**) Distribution of PC instantaneous firing regularity (LvR, **i**) and LvR s.d. (**j**) during quiet wakefulness (black, Qw, *n*=54 cells, *N*=47 mice) and during locomotion before (blue, Loc, *n*=54 cells, *N*=47 mice), and after light-evoked silencing of MLIs (green, Loc+Arch, *n*=16 cells, *N*=14 mice). *P<0.05, **P<0.01, two-tailed *t*-tests. (**k**) Normalized motion index (MI) aligned to the onset (left) and offset (right) of light stimulation (green). Grey traces represent the data from individual cells and black line represents smoothed average (*n*=11 and 14, respectively, *N*=21 mice). (**l**) Relative distributions of mice displaying no change, slowing or a complete halt in locomotion after onset (Light On) and offset (Light Off) of light stimulation (*n*=29, *N*=29 mice).

To confirm that the observed changes in d*V*_m_ directly influence PC spike output (Fig. 5g), we made somatic recordings before and after optogenetic silencing of MLIs (Fig. 6a). Blocking FFI resulted in a consistent increase in SSp firing rates across all PCs, irrespective of whether SSp firing rates were enhanced or suppressed before light stimulation (*n*=16 from *N*=14 mice; Fig. 6b). After cessation of the light stimulus SSp firing rates rapidly returned to levels observed before light activation (Fig. 6c) and light activation in the absence of Arch 3.0 expression did not affect the SSp firing frequency in PCs (Supplementary Fig. 8). Blocking inhibition also increased CS firing rates during both quiet wakefulness and locomotion (Supplementary Tables 6 and 7), consistent with enhanced SSp firing rates suppressing deep cerebellar nuclei-mediated inhibition of inferior olive activity (that is, disinhibition of olivary neurons leading to enhanced climbing fibre activity)^40^,^41^.

To estimate the ΔSSp firing rate ratio (excitation/inhibition) in individual PCs (see Methods), we again took advantage of the fact that PCs linearly transform excitatory and inhibitory inputs to perform a simple subtraction analysis^24^,^39^. We found that ΔSSp firing rate ratios were centred around unity, mirroring the Δd*V*_m_ ratios recorded in PC dendrites, and were not correlated with the baseline firing rate during quiet wakefulness^42^ (Fig. 6d–g). Thus, subtle changes in the balance between dendritic excitation and inhibition can generate robust, bidirectional changes in somatic SSp output during locomotion.

As expected, perturbing the excitation–inhibition balance by blocking FFI significantly decreased the coefficient of variation (CV) of interspike intervals in the majority of PCs, irrespective of whether CV changes were positive or negative in the absence of light stimulation (CV: Qw 0.53±0.03; Loc 0.66±0.05, *P*=5 × 10^−3^; Loc+Arch 0.32±0.02, *P*=1 × 10^−4^; *n*=16 cells from *N*=14 mice; Fig. 6h). To further examine how changes in excitation–inhibition affects the temporal pattern of PC SSp firing, we applied an analysis method termed local variability accounting for refractoriness (LvR; see Methods), which isolates the instantaneous firing regularity or irregularity of a neuron, independently of spike rate fluctuations^43^. Using this metric, we isolated changes in the temporal structure of PC spiking from rate-based fluctuations during behaviour. We found that locomotion increased the dispersion of LvR values—measured as the s.d. of the LvR distribution across PCs—when compared with quiet wakefulness (LvR s.d. Qw 55.4±8.2 × 10^−3^, Loc 78.5±7.5 × 10^−3^, *n*=54 cells from *N*=47 mice, *P*=2 × 10^−3^). The widening of the LvR distribution reflected an increase in the number of longer duration interspike intervals (ISIs) and irregularity of firing across the PC population. Blocking FFI produced a clear leftward shift in the LvR distribution and a reduction in the dispersion of LvR values (LvR s.d. Loc 78.5±7.5 × 10^−3^, Loc+Arch 18.7±3.8 × 10^−3^, *P*<1.0 × 10^−8^; Fig. 6i,j). To assess whether increased inhibitory input generated longer ISIs during locomotion, we plotted ISI length (ms) as a function of *V*_m_ distance to threshold (mV) for each ISI. We found that irrespective of the direction of the SSp firing rate change during locomotion ISI distributions became wider with the appearance of longer ISIs with more hyperpolarized *V*_m_ (Supplementary Fig. 9). Thus, locomotion-dependent modulation of the excitation–inhibition ratio in PCs appears necessary not only for generating bidirectional SSp modulation but also for enriching the repertoire of behaviour-related SSp patterns.

But is bidirectional SSp modulation necessary for normal locomotor control? Given that optogenetic silencing of MLIs abolished bidirectional SSp modulation and increased SSp regularity across the majority of PCs along the apex of lobule V (150 × 300 μm craniotomy with an estimated light penetration depth of ∼300 μm (ref. 44)), we investigated whether this manipulation affected self-paced locomotion. Light stimulation resulted in consistent changes in locomotor behaviour in ∼70% of the mice tested (7/29 reacted to ‘light on'; 10/29 mice reacted to ‘light off'; 4/29 mice reacted to ‘light on' and ‘light off'; 8/29 mice did not show any behavioural response), consisting of a slowdown or complete halt in locomotion after stimulation onset (slowdown 31.0%, *n*=9/29; stop 6.9%, *n*=2/29) and offset (slowdown 37.9%, *n*=11/29; stop 10.3%, *n*=3/29; Fig. 6k,l). Light-evoked changes had an average onset latency of 161±23 ms and offset latency of 111±24 ms. The observed behavioural effects were similar in magnitude and duration to those evoked by direct optogenetic stimulation of PCs using channelrhodopsin^45^, suggesting that both manipulations disrupt the same downstream motor-related pathways. Importantly, changes in locomotor behaviour were not due to the nonspecific effects of light stimulation, as mice did not react when stimulated in the absence of Arch 3.0 expression (Supplementary Fig. 8). Together, our findings suggest that the fine balance between dendritic excitation and inhibition provides a sensitive ‘push-pull' mechanism to generate the bidirectional modulation of PC SSp output necessary for normal locomotor behaviour.

## Discussion

In this study, we investigated the cellular mechanisms underpinning locomotion-dependent bidirectional modulation of PC SSp output by performing somatic and dendritic intracellular recordings *in vivo*, optogenetic silencing of MLIs and quantitative behavioural analysis. Our data reveal three main findings. First, we show that GCs generate a sustained depolarization of PC dendrites during locomotion, the effects of which are counterbalanced by variable levels of inhibitory input from MLIs. Second, we demonstrate that locomotion-dependent modulation of the balance between excitation and inhibition generates depolarizing or hyperpolarizing dendritic *V*_m_ changes that linearly transform into bidirectional modulation of PC SSp output. Finally, we show that perturbing the excitation–inhibition balance by optogenetic silencing of MLIs abolishes bidirectional firing rate changes and enhances the rate and regularity of SSps leading to disrupted locomotor function.

To directly investigate how the dendritic excitation–inhibition balance shapes PC SSp output during motor behaviour, we took advantage of the fact that the somatodendritic structure of PCs, with primary and secondary dendrites measuring ∼3–8 μm in diameter, makes them amenable to intracellular recording *in vivo*. By performing the first intracellular dendritic recordings from PCs in awake behaving mice, we were able to simultaneously monitor changes in d*V*_m_ and dendritic spikelets—that is, passive backpropagation of SSps from the soma to the dendrites—to characterize PC input–output transformations during self-paced locomotion. We found that the locomotion-dependent recruitment of feedforward excitatory and inhibitory inputs generated both depolarizing and hyperpolarizing changes in d*V*_m_ that linearly transformed into bidirectional modulation of PC SSp output. The d*V*_m_ changes measured in our recordings reflect the dynamic, time-varying balance between excitation, inhibition and powerful intrinsic conductances present in the dendrite^6^,^7^,^8^,^9^,^46^. Experimental and modelling studies have suggested that excitatory and inhibitory conductances exert a partial voltage-clamp on the dendrite, where the dendritic membrane potential remains close to the clamping voltage dictated by the excitation/inhibition ratio. In this regard, subtle changes in either excitation or inhibition can significantly alter the dendritic membrane potential and firing rate of PCs^18^,^19^,^46^. Consistent with these predictions, we show that a 2–3 mV change in PC d*V*_m_ resulted in a 50–100% change in SSp firing rate when measured in the dendrite (dSSps) or soma (SSps). Disrupting the excitation–inhibition balance by blocking FFI increased the magnitude of the Δd*V*_m_ and SSp firing rate change without affecting the linearity of the relationship, consistent with PCs using a simple linear summation algorithm to integrate synaptic inputs and regulate SSp output during locomotion^24^,^39^. Although our results describe the excitation/inhibition balance in terms of direct feedforward input to the dendrites, basket cell inhibition and ascending GC axon inputs onto the soma could in principle influence d*V*_m_ changes via backpropagation into the dendrites^21^,^47^,^48^,^49^. Unfortunately, due to technical reasons, we were unable to differentiate between the effects of stellate cell versus basket cell inputs, or GC ascending versus parallel fibre inputs on PC input-output transformations. Although our findings provide the first detailed description of how dendritic excitation and inhibition combine to shape PC *V*_m_ dynamics during motor behaviour, SSp output involves complex interactions between afferent synaptic input and large voltage-dependent conductances in the soma and dendrites^6^,^7^,^8^,^9^,^46^; therefore, further work will be necessary to elucidate the role of intrinsic voltage-dependent conductances in regulating PC output during locomotion.

A signature of PC population activity during locomotion is the bidirectional modulation of SSp firing rates^1^,^2^,^3^,^4^,^5^. During locomotion, PCs display enhanced or suppressed activity, suggesting that sensorimotor information can equally be encoded by an increase or decrease in SSp firing rate. In the sagittal orientation, PCs receive strong excitation from GCs positioned medially and pure inhibition from GCs positioned >300 μm lateral^24^,^50^, thus providing an anatomical substrate for the bidirectional modulation of PC activity during behaviour. To test which of our three simple input models could explain bidirectional SSp modulation during self-paced locomotion (Fig. 1), we examined behaviour-related activity along the GC–PC and GC–interneuron–PC pathways. At rest, PCs and MLIs receive a constant barrage of GC input that in principle could be bidirectionally modulated if subsets of GCs increased their firing rates, while others reduced their output. Given the high rate of convergence of GC inputs to downstream neurons, a slight reduction in individual firing rates could result in a significant reduction in FFE and firing rates of PCs and MLIs. However, our GC and PC dendritic recordings clearly show that as a population GCs provide sustained FFE during locomotion^5^,^22^, discounting input ‘model 1' (that is, bidirectional modulation of excitation with enhanced inhibition). Since all MLIs displayed an increase in firing rate during locomotion, local feedback inhibition in the molecular layer appears insufficient to dampen GC-mediated FFE of MLIs. The absence of reciprocal MLI firing rates during locomotion suggests that input ‘model 2' (that is, enhanced excitation with bidirectional modulation of inhibition) is unlikely to underpin the changes in PC d*V*_m_. Instead, our data are consistent with feedforward input ‘model 3' in which PCs receive both enhanced dendritic excitation and inhibition during locomotion, the balance of which contributes to a sensitive ‘push-pull' mechanism that drives bidirectional modulation of PC SSp output (Fig. 1).

One unexpected observation was that GCs displayed a ∼70-fold increase in spiking activity during locomotion that transformed into a modest twofold increase in charge transfer in MLIs. Although the reasons for this disparity remain unclear, one possible explanation is that MLIs receive a high rate of miniature (action potential independent) parallel fibre EPSCs during quiet wakefulness—that would be unaffected by enhanced GC activity during locomotion—and action potential-dependent input from a limited number of GCs. Alternatively, high-frequency burst firing of parallel fibres during locomotion can induce short-term presynaptic plasticity mediated by GABA_A_, GABA_B_ and mGluR_4_ receptors, leading to a significant reduction in parallel fibre synaptic transmission^51^,^52^. Further studies will be required to investigate how parallel fibre plasticity affects the fidelity of information transmission during behaviour.

Cerebellar PCs generate two different types of spikes: CSs reflecting activation of climbing fibre inputs and SSps reflecting intrinsic spike generation that can be modulated by synaptic input. The rate of SSp and CS activity is in general reciprocal in nature—that is, when the SSp frequency increases CS rates decrease and vice versa^13^,^53^,^54^. Until recently, the network mechanism underpinning SSp and CS reciprocity remained unresolved due to the difficulty of modulating rhythmic CS input without a concomitant effect on SSp firing rates. By selectively rerouting climbing fibres from an ipsilateral projection to a contralateral projection, Badura *et al*.^34^ were able to show that climbing fibres drive reciprocal SSp–CS firing rates via recruitment of FFI from MLIs. Accordingly, our data demonstrate that a significant proportion of PCs display SSp–CS reciprocity during quiet wakefulness and self-paced locomotion (Fig. 1). By abolishing FFI, GC excitation dominates, increasing PC SSp rates (Fig. 6). This in turn suppresses deep cerebellar nuclei-mediated inhibition of inferior olive activity (that is, disinhibition of olivary neurons), leading to a coincident increase in SSp and CS firing rates (Supplementary Tables 6 and 7)^55^. Thus, it will be of interest to assess the extent to which PF- versus CF-mediated recruitment of FFI regulates PC SSp firing rates during quiet wakefulness and locomotion.

But are bidirectional SSp firing rate changes necessary for normal locomotor behaviour? Extracellular recordings from PCs have demonstrated reciprocal firing rate changes in cats trained to perform constant speed locomotion on a linear treadmill, where changes in firing rate correlate with different phases of the step cycle^1^. More recently, optogenetic activation of PC ensembles has been shown to orchestrate complex multi-joint movements in mice, where light stimulation evoked a slowing or complete arrest of self-paced locomotion^45^. The fact that bidirectional changes in dendritic *V*_m_ generated reciprocal PC firing rates, which when abolished, disrupted locomotor function, confirm their importance for normal motor control. Silencing MLIs evoked strong behavioural responses that were time-locked to both the onset and offset of optogenetic light stimulation, consistent with increased PC activity suppressing deep cerebellar nuclei (DCN) output during light activation followed by a rebound excitation of DCN neurons after stimulus cessation^45^,^56^,^57^,^58^. Although our findings provide a mechanistic link between the dendritic excitation–inhibition balance in PCs, bidirectional SSp modulation and normal locomotor function, further experiments will be required to understand how time-varying output from the cerebellar cortex—via the DCN—engages downstream motor areas to coordinate simple and complex cerebellum-dependent motor behaviours. Given the consistent cytoarchitecture across the cerebellar cortex, our findings may generalize to other areas of the cerebellum where differential recruitment of feedforward excitatory and inhibitory circuits may provide a common dynamic ‘push-pull' mechanism to shape PC input–output transformations during motor behaviour.

## Methods

### Animal care and housing

Male 6–10-week-old, C57BL/6 or *Nos1Cre*^37^ mice, two to six animals per cage, were housed on a reversed 12 h light/dark cycle. Food and water were available *ad libitum*. All animal procedures were approved by the University of Edinburgh local ethical review committee and performed under licence from the UK Home Office in accordance with the Animal (Scientific Procedures) Act 1986.

### Surgeries

To perform *in vivo* awake recordings, a small lightweight headplate (0.75 g; www.DuguidLab.com) was attached to the skull under 1.5% isoflurane anaesthesia using cyanoacrylate adhesive and dental acrylic. The headplate formed a recording well that was 3 mm in diameter in which a craniotomy was performed at least 24 h after recovery. The craniotomy (∼150 × 300 μm) was positioned directly above lobule V of the cerebellum (2.5 mm posterior to lambda, 0.75 mm lateral to midline) and the dura was removed. The craniotomy was sealed with agar (1.5%) and Kwik-Cast sealant (WPI, Europe), and mice were returned to the home cage for ∼1 h to recover from anaesthesia before recording commenced.

### *In vivo* electrophysiology

Mice were habituated to the head restraint and experimental setup for 30–60 min before each recording session. Head-restrained mice were free to run, walk or sit on the cylindrical treadmill. Current and voltage-clamp recordings were performed at 50–300 μm from pial surface for MLIs, 250–350 μm for PCs and >350 μm for GCs using a Multiclamp 700B amplifier (Molecular Devices, USA). The data were filtered at 6–10 kHz and digitized at 10–20 kHz using PClamp 10 software in conjunction with a DigiData 1440 DAC (Molecular Devices). No bias current was injected during recordings and the membrane potential was not corrected for junction potential. Resting membrane potentials were recorded immediately after ‘break-in' and series resistances ranged between 20 and 50 MΩ. Series resistances were compensated by ∼50% when recording EPSC in MLIs. *In vivo* external solution contained (in mM) 150 NaCl, 2.5 KCl, 10 HEPES, 1.5 CaCl_2_ and 1 MgCl_2_, (pH 7.3). Patch pipettes (5–8 MΩ) were filled with (in mM) 135 K-gluconate, 7 KCl, 10 HEPES, 10 sodium phosphocreatine, 2 MgATP, 2 Na_2_ATP and 0.5 Na_2_GTP (pH 7.2, 285–295 mOsm), and 1–2 mg ml^−1^ biocytin was added before recording. Excitatory synaptic input was measured in MLIs by voltage clamping at −70 mV. At the end of the recording, mice were transcardially perfused with 4% paraformaldehyde and the brain removed for *post hoc* immunohistochemistry.

### Virus injection and optogenetic stimulation

*Nos1Cre* mice^37^ were anaesthetized with 1.5% isoflurane, and two burr holes (50 × 50 μm) were created 400–700 μm apart above lobule V (2.5 mm posterior to lambda, 0.75 mm lateral to midline). rAAV2-EF1a-DIO-eArch3.0-eYFP (UNC VectorCore; 300 nl per injection site) was injected using a Picospritzer at a depth of 100–350 μm from the pial surface through a pulled glass micropipette. After injection, mice were allowed to recover for 8–10 days to ensure sufficient expression of Arch 3.0. Photostimulation of Arch 3.0 was achieved using a custom designed optic fibre dual lens coupling (1 mm diameter; 0.48 numerical aperture; Doric lenses) and 532 nm, 200 mW single wavelength laser (Laserlands, China). For Arch 3.0 activation, laser intensity was 70–80 mW mm^−2^ when measured as the output from the lens. Given that the lens housing was positioned ∼10 mm from the craniotomy and that the light had to transition from air to solution then to brain, the actual irradiance 50–300 μm from the pial surface will be significantly reduced. For technical reasons, we were unable to directly measure irradiance in the molecular layer. To ensure that light stimulation alone did not evoke cellular responses (that is, via heat-mediated effects), control experiments were performed in *Nos1Cre* mice in the absence of Arch 3.0 virus (Supplementary Fig.7). Mice were habituated to the head restraint and experimental setup for 1 h before each recording session and the laser switched on/off—while blocking the craniotomy with silicon sealant—to check for behavioural responses. At the point that mice did not react to noises mimicking experimental procedures or light stimulation, they were deemed fully habituated. Laser stimulation was 3 s in duration during quiet wakefulness and was manually controlled during self-paced locomotion. Light stimulation commenced ∼2–5 s after locomotion onset and remained on for ∼2–4 s. Light-evoked changes in locomotor behaviour were assessed by analysing the MI 500 ms before and 1,000 ms after the onset/offset of light stimulation. The threshold for light-evoked behavioural responses was set at >2 × the s.d. of the MI before the onset/offset of light stimulation. If the MI decreased below our defined threshold but above the MI levels observed during quite wakefulness, then the behavioural change was classified as ‘slowing down'. If, however, the MI decreased to levels similar to that observed during quite wakefulness and lasted for >200 ms, this signified a complete halt in locomotion (stop).

### Immunohistochemistry

After transcardial perfusion, individual brains were post-fixed overnight in 4% paraformaldehyde and parasagittal sections (60 μm) were cut using a vibratome (Leica VT1000S). For reconstruction of neuronal morphology, sections were first incubated in blocking solution for 2 h (10% normal goat serum, 0.5% Triton X-100 in 0.01 M PBS), and then incubated overnight in streptavidin AlexaFluor-488 or 568 (1:1,000, Molecular Probes). To verify selective expression of Arch 3.0 in MLIs, sections from transfected brains were first incubated in blocking solution for 2 h, and then incubated in mouse anti-parvalbumin (1:2000, Swant, Switzerland) primary and anti-mouse AlexaFluor-568 secondary antibodies (1:1,000, Molecular Probes). All antibodies were diluted in carrier solution (0.01 M PBS, 2% normal goat serum and 0.5% Triton X-100) and incubated overnight at room temperature. Slices were mounted and *z* stacks acquired using a Nikon A1R FLIM confocal microscope ( × 20 and × 40 objectives; Nikon, Europe). Quantification of Arch 3.0 and parvalbumin co-localization was achieved by manual counting of cells in 1 μm optical sections focused along the apex of lobule V (*n*=16 slices from *N*=4 mice).

### Motion index and locomotion

All movements (positioning, grooming and locomotion) were captured using an elevated, front-mounted digital camera (60 f.p.s.) synchronized with each electrophysiological recording. An optical encoder was used to capture movement of the treadmill and to calculate the speed of locomotion. A motion index (MI) was calculated from successive video frames: MI_f_=Σ^*N*^_*i*=1_(*c*_*f*+1,*i*_−*c*_*f*,*i*_)^2^, where *c*_f,i_ is the grayscale level of the pixel *i* of the region of interest (ROI) in frame *f*. In each recording, we selected a full frame ROI that included the trunk, face, forelimbs and hindlimbs from the front elevation view. Given that MI was calculated as pixel changes during successive video frames, this provided a sensitive measure to detect any movement during movement preparation, onset or steady-state locomotion. Locomotion was defined as periods of walking or running that lasted for >3 s checked by visual inspection of each video. The threshold for locomotion onset was set at 2 × the s.d. of the MI during periods of quiet wakefulness. The average length of each bout of locomotion was 11.9±0.6 s (range: 4–28.5 s). Cells recorded from mice that did not engage in self-paced voluntary locomotion were excluded from the comparison analysis of quiet wakefulness versus locomotion but were used in describing the basic firing properties of each cell type.

### Data analysis

Data analysis was performed with MatLab and custom-written macros in Igor Pro 6 (Wavemetrics, USA). Input resistances were calculated from current responses during 400 ms step voltage (−20 mV) injections. Synaptic events were detected using an amplitude threshold algorithm (TaroTools, custom-written macro in Igor Pro 6), where the threshold for detection was set at 2 × the s.d. of the noise (typically the threshold was set at 10–15 pA for MLIs).

The location of dendritic recordings was estimated based on known depth of the PC layer from cell-attached or whole-cell recordings in the same preparation. Simple and CSs were detected automatically (TaroTools) and were manually verified. Interspike intervals preceding and following CSs were removed from SSps analysis and CSs were blanked when calculating d*V*_m_.

Charge transfer of MLIs was calculated as the time-integral of the current in a 20 ms sliding window after subtraction of the holding current. Baseline current was estimated based on the lowest current amplitude minimum in each individual trace. Membrane potential spike threshold was computed as the second or third derivative of the voltage trace recorded in MLIs and PCs, respectively. Average membrane potential was calculated after clipping spikes from threshold. The interspike modal *V*_m_ of PCs was calculated from 1.77±0.12 ms after the peak of each spike to the threshold of the following spike using a custom-written macro in Igor Pro 6. Burst analysis of GCs was conducted using a custom-written macro in Igor Pro 6, where bursts were defined as having at least three spikes with an intra-bust interval of 30 ms or less. To estimate the correlation between firing rate/charge transfer and locomotion Pearson correlation, coefficients were calculated between frequency/charge and MI from the whole trace (90 s) using a 20 ms bin size.

To investigate the SSp–CS firing rate reciprocity, we calculated the mean SSp frequency during each CS interspike interval (that is, instantaneous CS frequency). SSp frequency was plotted as a function of CS frequency and SSp–CS correlation coefficients were defined by applying linear regression analysis. To explore the relationship between firing rate/d*V*_m_ or charge and MI in PCs and MLIs, exponential functions were fitted in Igor Pro 6 (y_0_+Aexp(- invTau * x) to the binned (200 ms bin size) raw data of frequency/charge versus MI in individual cells (Supplementary Fig. 4). Data values were normalized based on the minimum and maximum value of each dataset.

To estimate the effect of FFI on the d*V*_m_, we subtracted the Δd*V*_m_ during locomotion in the presence of light stimulation (Δd*V*_m_ Loc+Arch) from the Δd*V*_m_ during locomotion (Δd*V*_m_ Loc), taking into account the effect of baseline excitation during quiet wakefulness (Δd*V*_m_ Qw+Arch). Thus, the estimated effect of inhibition during locomotion was calculated using the equation Δd*V*_m_ Loc−(ΔdV_m_ Loc+Arch)+(ΔdV_m_ Qw+Arch). Given that we did not record the effects of Arch stimulation during Qw and Loc in each cell, we instead used an average Δd*V*_m_ Qw+Arch value (2.04±0.48 mV, *n*=6). Similarly, to estimate the effect of FFI on the somatic firing rate of PCs, we adopted the same strategy as above using the equation ΔSSp Loc−(ΔSSp Loc+Arch)+(ΔSSp Qw+Arch). For this set of experiments, the ΔSSp Qw+Arch for each individual cell was used. To estimate the effect of excitation during locomotion, we subtracted the baseline excitatory component (Δd*V*_m_ or ΔSSp Qw+Arch) from the Δd*V*_m_ or ΔSSp during locomotion in the presence of light stimulation (that is, (Δd*V*_m_ or ΔSSp Loc+Arch)−(Δd*V*_m_ or ΔSSp+Arch)). The ratio of effects of excitation (Loc+Arch)−(Qw+Arch) versus inhibition (Loc−(Loc+Arch)+(Qw+Arch)) was calculated using absolute values of Δd*V*_m_ or ΔSSp change (that is, negative values were converted to positive).

The firing regularity/irregularity of PCs (LvR) was defined by the equation:


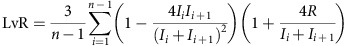


where *I*_*i*_ and *I*_*i*+1_ are the *i*th and *i*+1th ISIs, and *n* is the number of ISIs. *R* is a refractoriness constant characteristic to each cell type (*R* was set at 3 ms for PCs)^43^. Error bars indicate mean±s.e.m. unless stated otherwise. To determine statistical significance, two-tailed *t*-tests were used if the distribution of the samples passed the normality test. For GC spike frequency analysis, a Wilcoxon matched-pairs signed-rank non-parametric test was used. *P*<0.05 was considered significant (*P<0.05, **P<0.01).

### Data availability

The data that support the findings of this study are available from the corresponding author on reasonable request.

## Additional information

**How to cite this article:** Jelitai, M. *et al*. Dendritic excitation–inhibition balance shapes cerebellar output during motor behaviour. *Nat. Commun.* **7**, 13722 doi: 10.1038/ncomms13722 (2016).

**Publisher's note:** Springer Nature remains neutral with regard to jurisdictional claims in published maps and institutional affiliations.

## Supplementary Material

Supplementary Information Supplementary Figures and Supplementary Tables.
