# Supplementary material for: Dendritic excitation–inhibition balance shapes cerebellar output during motor behaviour
Source: Nat Commun. 2016 Dec 15;7:13722. doi: 10.1038/ncomms13722 (PMC5172235; doi:10.1038/ncomms13722)
Supplement: Supplementary Information — Supplementary Figures and Supplementary Tables. [file ncomms13722-s1.pdf]

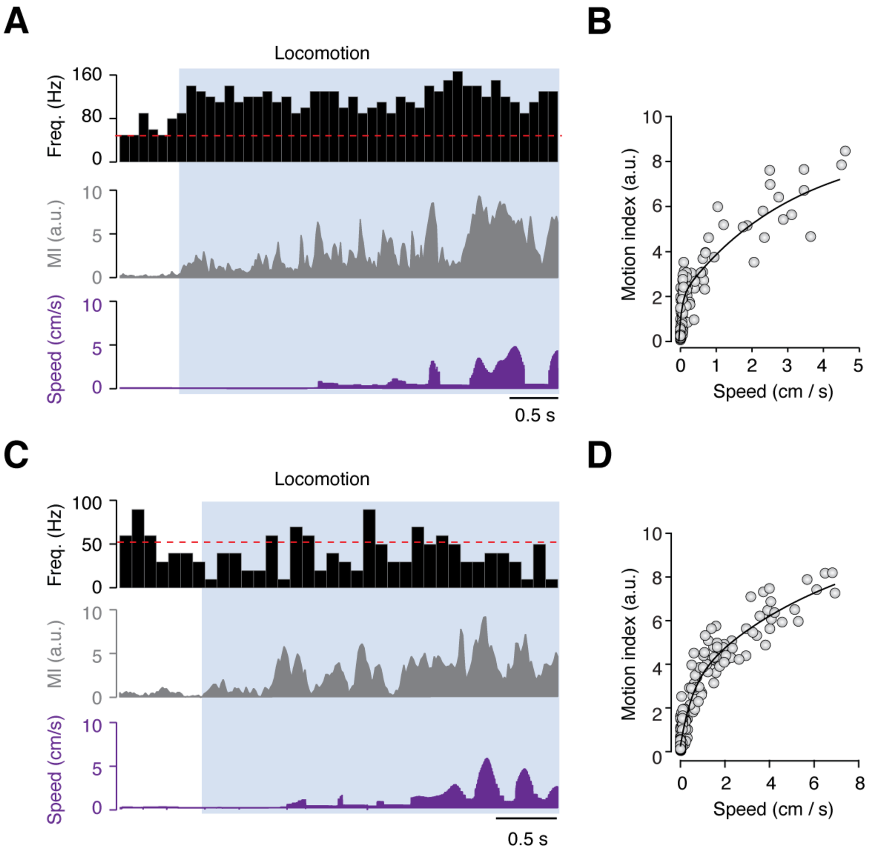

Supplementary Fig. 1. Motion index versus speed during locomotion.

(**A**) Representative Purkinje cell spike frequency histogram (upper panel, bin size = 100 ms), motion index (middle panel) and treadmill speed (lower panel) during quiet wakefulness and voluntary locomotion (blue shading). Red dashed line indicates average frequency during quiet wakefulness. Note the delay between M.I. and speed onset (i.e. speed lags motion index by >1 second).

(**B**) Change in motion index as a function of increasing speed (bin size = 200 ms) generated from the same cell as shown in panel A. Black line represents exponential fit to the data.

(**C**) Representative Purkinje cell spike frequency histogram (upper panel, bin size = 100 ms), motion index (middle panel) and treadmill speed (lower panel) during quiet wakefulness and voluntary locomotion (blue shading). Red dashed line indicates average frequency during quiet wakefulness. Note the delay between M.I. and speed onset (i.e. speed lags motion index by >1 second).

(**D**) Change in motion index as a function of increasing speed (bin size = 200 ms) generated from the same cell as shown in panel C. Black line represents exponential fit to the data.

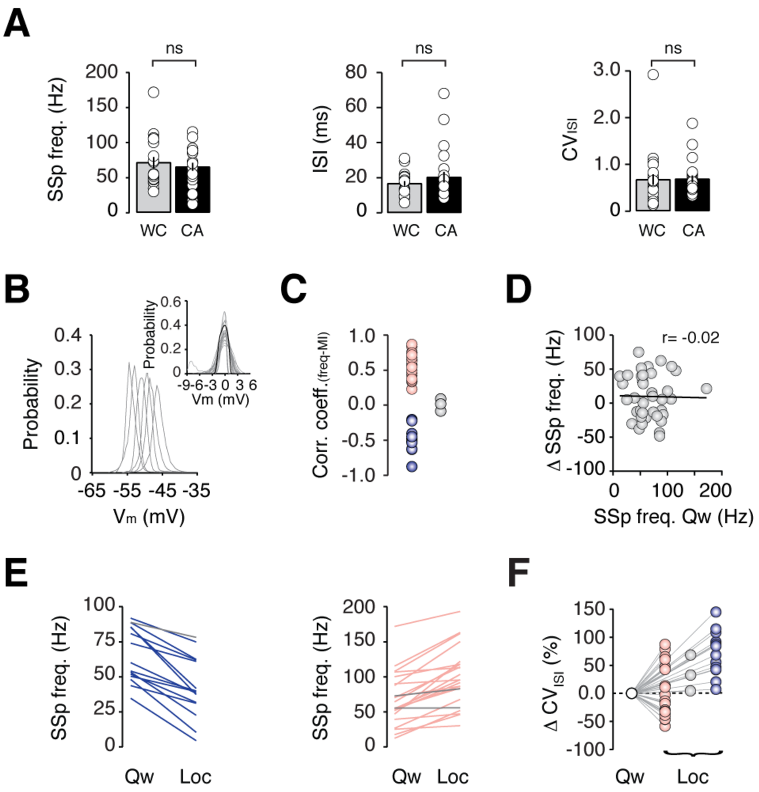

Supplementary Fig. 2. Purkinje cell SSp firing properties.

(**A**) Average quiet wakefulness firing rate, interspike interval (ISI) and coefficient of variation (CV) of SSp ISIs in Purkinje cells measured via whole-cell (WC, *n* = 24 from N = 24 mice) and cell-attached (CA, *n* = 24 from N = 20 mice) recording configurations. Bars represent mean ± s.e.m. Open circles represent data from individual Purkinje cells, ns = not significant, two-tailed *t* test.

(**B**) Typical membrane potential (V_m) distributions of six Purkinje cells during quiet wakefulness. Inset: peak aligned membrane potential (V_m) distribution of all Purkinje cells where gray lines denote data from individual cells and the black line highlights a single typical V_m distribution (*n* = 24 cells from N = 24 mice).

(**C**) Pearson correlation coefficient between firing frequency and motion index (bin size = 20 ms). Pink symbols represent Purkinje cells with a significant positive correlation (*n* = 21/38, p < 0.01), blue symbols represent Purkinje cells with a significant negative correlation (*n* = 14/38, *P* < 0.001) and gray symbols represent Purkinje cells with no significant correlation (*n* = 3/38 from N = 33 mice).

(**D**) Change in simple spike firing rate (ΔSSp) during locomotion as a function of increasing firing rate during quiet wakefulness. Note the absence of any correlation (*r* = -0.02, *P* = 0.914, *n* = 38 cells from N = 33 mice). Gray circles represent data from individual Purkinje cells, black line is a linear fit to the data.

(**E**) Average firing rate of Purkinje cells during quiet wakefulness (Qw) and locomotion (Loc). Data have been separated into Purkinje cells that displayed decreased (blue, *n* = 14/38 cells), increased (pink, *n* = 21/38 cells) or no change (gray, *n* = 3 cells) in SSp firing rate during locomotion.

(**F**) Average change in the coefficient of variation of interspike intervals (CV_{ISI}) in Purkinje cells during quiet wakefulness (Qw) and locomotion (Loc). Coloured symbols represent Purkinje cells that displayed an increase (pink), decrease (blue) or no change (gray) in firing rate during locomotion (*n* = 38 cells from N = 33 mice).

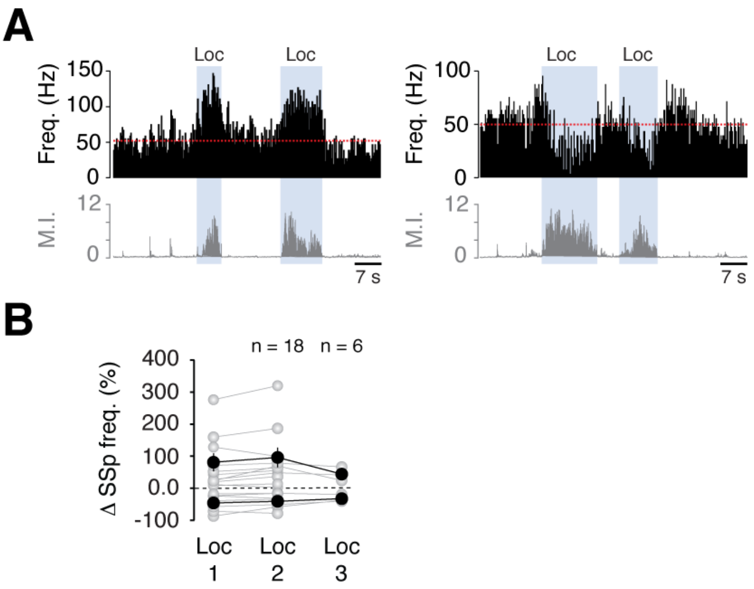

Supplementary Fig. 3. PC firing rates across successive locomotion bouts.

(**A**) Representative SSp frequency histograms (bin size = 250 ms) for Purkinje cells that displayed an increase (left hand panel) or decrease (right hand panel) in SSp frequency across 2 successive bouts of locomotion (blue shading). Motion index (M.I., dark gray) shows the onset and duration of each locomotion bout. Red dashed line indicates the average frequency during quiet wakefulness.

(**B**) Average Purkinje cell SSp frequency changes during 2 (*n* = 18 cells from N = 17 mice) or 3 (*n* = 6 cells from N = 6 mice) successive bouts of locomotion. Grey symbols and connecting lines represent data from individual Purkinje cells, black symbols represent mean ± s.e.m.

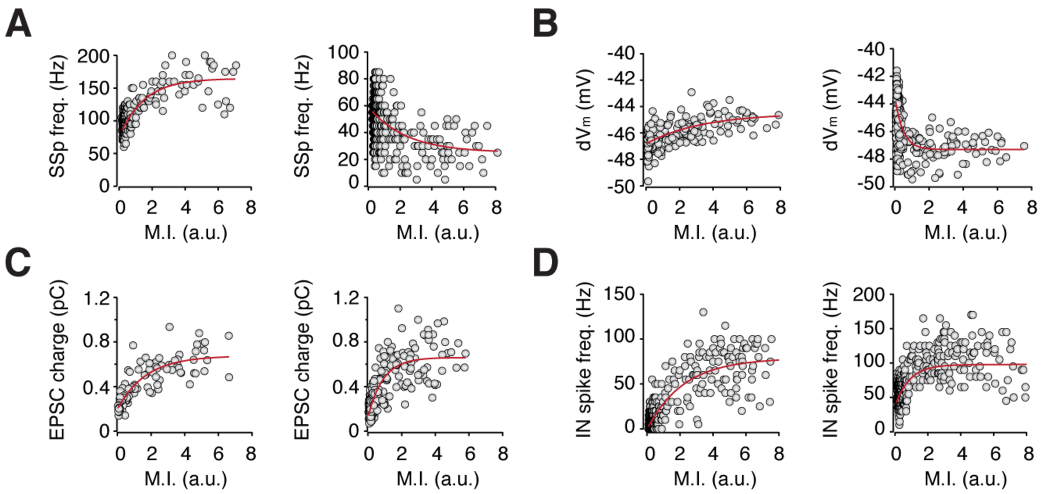

Supplementary Fig. 4. PC SSp firing rate, dV_m and IN charge transfer and firing rate changes as a function of increasing motion index.

(**A**) Average change in Purkinje cell SSp firing rates as a function of increasing movement (M.I. = motion index). The left hand panel represents a Purkinje cell that increased SSp frequency during movement, the right hand panel represents a Purkinje cells that decreased SSp firing rate. Circles represent the average value of SSp frequency and M.I. per bin (200 ms bin size). Red lines represent exponential fits to the data.

(**B**) Average change in Purkinje cell dendritic membrane potential as a function of increasing movement (M.I. = motion index). The left hand panel represents a Purkinje cell where the dV_m depolarized during movement, while the right hand panel represents a Purkinje cell where the dV_m hyperpolarized. Circles represent the average value of dV_m and M.I. per bin (200 ms bin size). Red lines represent exponential fits to the data.

(**C**) Average change in EPSC charge transfer as a function of increasing movement (M.I. = motion index) in two representative molecular layer interneurons. Circles represent the average value of charge transfer and M.I. per bin (200 ms bin size). Red lines represent exponential fits to the data.

(**D**) Average change in interneuron spike frequency as a function of increasing movement (M.I. = motion index) in two representative molecular layer interneurons. Circles represent the average value of IN spike frequency and M.I. per bin (200 ms bin size). Red lines represent exponential fits to the data.

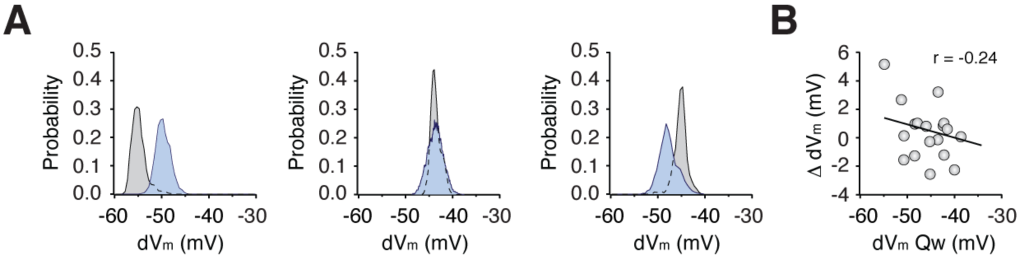

Supplementary Fig. 5. PC dV_m changes during locomotion.

(**A**) Representative Purkinje cell dV_m distributions showing a depolarization (left), no change (middle) and hyperpolarization (right) during the transition from quiet wakefulness (grey) to locomotion (blue).

(**B**) Locomotion-induced dendritic V_m changes as a function of quiet wakefulness dV_m. Grey circles represent data from individual dendritic recordings and black line is a linear fit to the data (r = -0.24, P = 0.31, n = 19 from N = 17 mice).

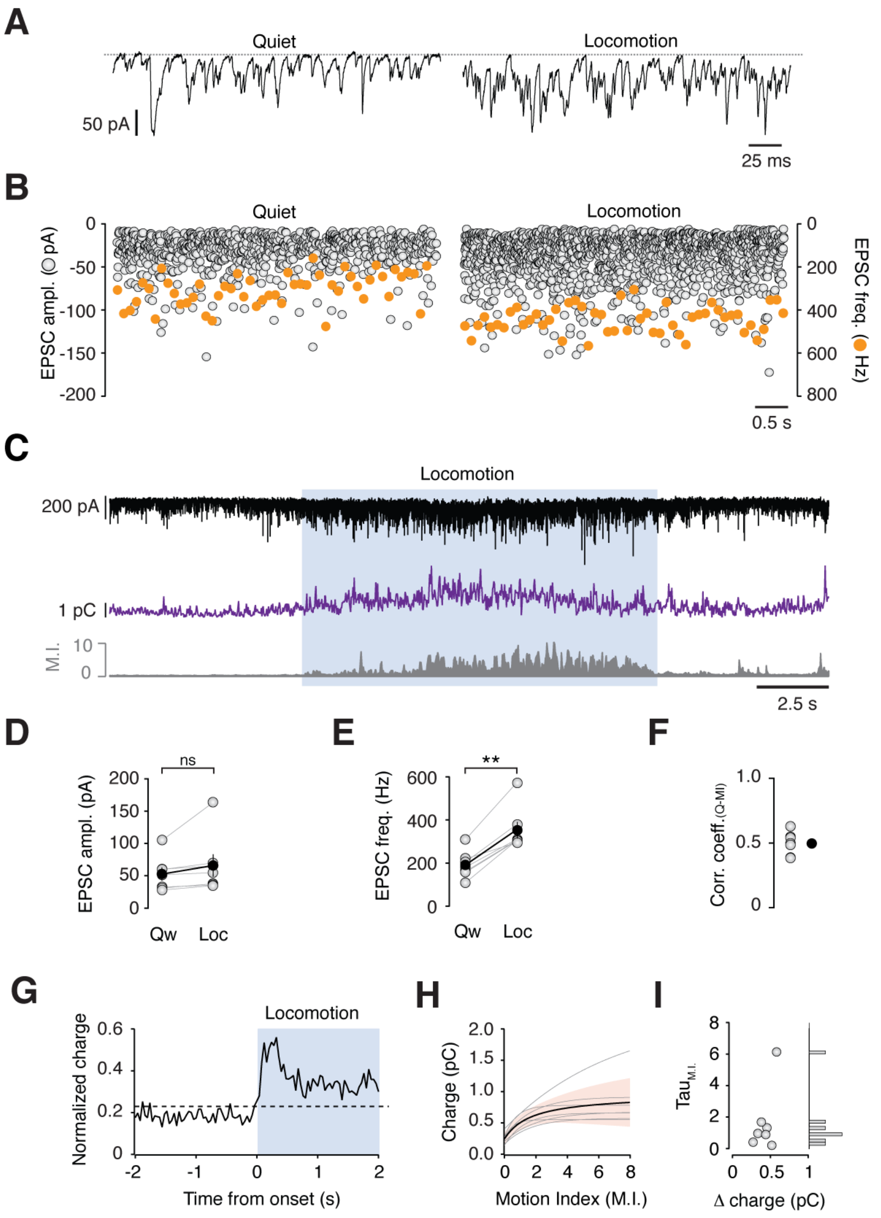

Supplementary Fig. 6. Parallel fiber input to MLIs during locomotion.

(**A**) Representative current trace recorded in a molecular layer interneuron during quiet wakefulness (left panel) and locomotion (right panel).

(**B**) Individual EPSC amplitudes (gray circles) and average EPSC frequency (orange circles, bin size = 100 ms) recorded at -70 mV during quiet wakefulness (left panel) and locomotion (right panel).

(**C**) Representative current trace (upper panel), corresponding charge transfer (middle panel) and motion index (lower panel) recorded from a molecular layer interneuron during quiet wakefulness and voluntary locomotion (blue shading).

(**D-E**) Average EPSC amplitude (D) and frequency (E) recorded during quiet wakefulness (Qw) and locomotion (Loc). Grey symbols and connecting lines represent data from individual interneurons, black symbols represent mean ± s.e.m., ns = not significant, $^{**}P < 0.01$, two-tailed *t* test ($n = 7$ cells from N = 6 mice).

(**F**) Pearson correlation coefficient reflecting the linearity of the relationship between EPSC charge transfer and motion index (bin size = 20 ms). Filled grey symbols represent data from individual interneurons and filled black symbol represents mean ± s.e.m. ($n = 7$ cells from N = 6 mice).

(**G**) Normalized charge transfer aligned to the onset of locomotion (blue shading, bin size = 20 ms, $n = 10$ bouts of locomotion from N = 7 cells). Dashed line represents 2 x standard deviation of the mean charge transfer during quiet wakefulness.

(**H**) Average charge transfer as a function of increasing movement (motion index). Thin grey lines represent exponential fits to the data in individual cells, black line represents the average across all cells and pink shading the standard deviation of the mean ($n = 7$ cells from N = 6 mice).

(**I**) Distribution of $Tau_{M.I.}$ values taken from the exponential fits shown in panel (H).

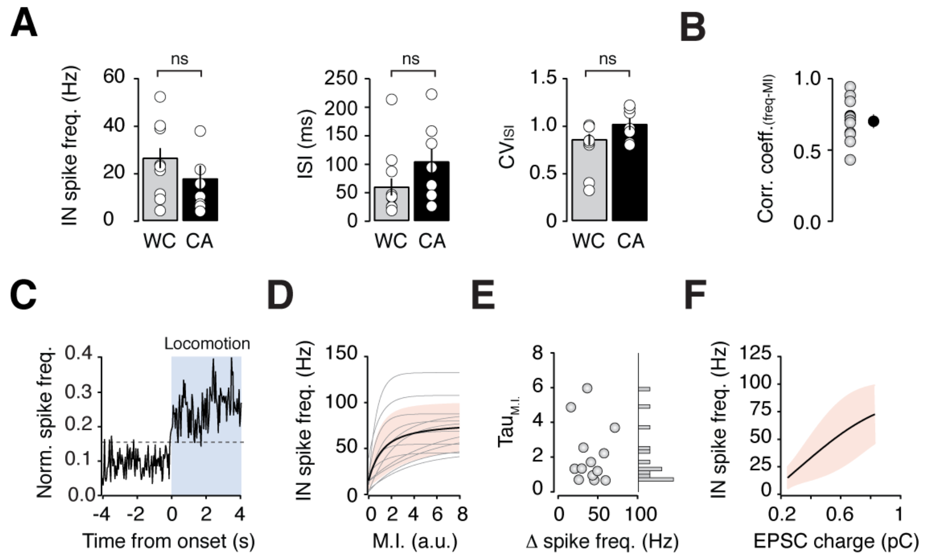

Supplementary Fig. 7. MLI firing properties during quiet wakefulness and locomotion.

(**A**) Average quiet wakefulness firing rate, interspike interval (ISI) and coefficient of variation (CV) of ISIs in interneurons measured via whole-cell (WC, *n* = 13 from N = 13 mice) and cell-attached (CA, *n* = 10 from N = 9 mice) recording configurations. Bars represent mean ± s.e.m. Open circles represent data from individual interneurons. ns = not significant, two-tailed *t* test.

(**B**) Pearson correlation coefficient reflecting the linearity of the relationship between firing frequency and motion index (bin size = 20 ms). Grey symbols represent data from individual interneurons, black symbol represents mean ± s.e.m. (*n* = 13 cells from N = 13 mice).

(**C**) Normalized firing frequency aligned to the onset of locomotion (blue shading, bin size = 20 ms, *n* = 16 bouts of locomotion from N = 13 cells). Dashed line represents 2 x standard deviation of the mean firing frequency during quiet wakefulness.

(**D**) Average change in interneuron firing frequency as a function of increasing movement (motion index). Thin gray lines represent exponential fits to the data in individual cells, black line represents the average across all cells and pink shading the standard deviation of the mean (*n* = 13 cells from N = 13 mice).

(**E**) Distribution of $Tau_{M.I.}$ values taken from the exponential fits shown in panel (D).

(**F**) Input-output relationship generated by plotting the average EPSC charge transfer vs firing frequency for each binned motion index value (200 ms bin size) (i.e. combining input and output data from different populations of cells shown in Figure 3H and Figure 3L). Black line represents the average, pink shading represents standard deviation of the mean.

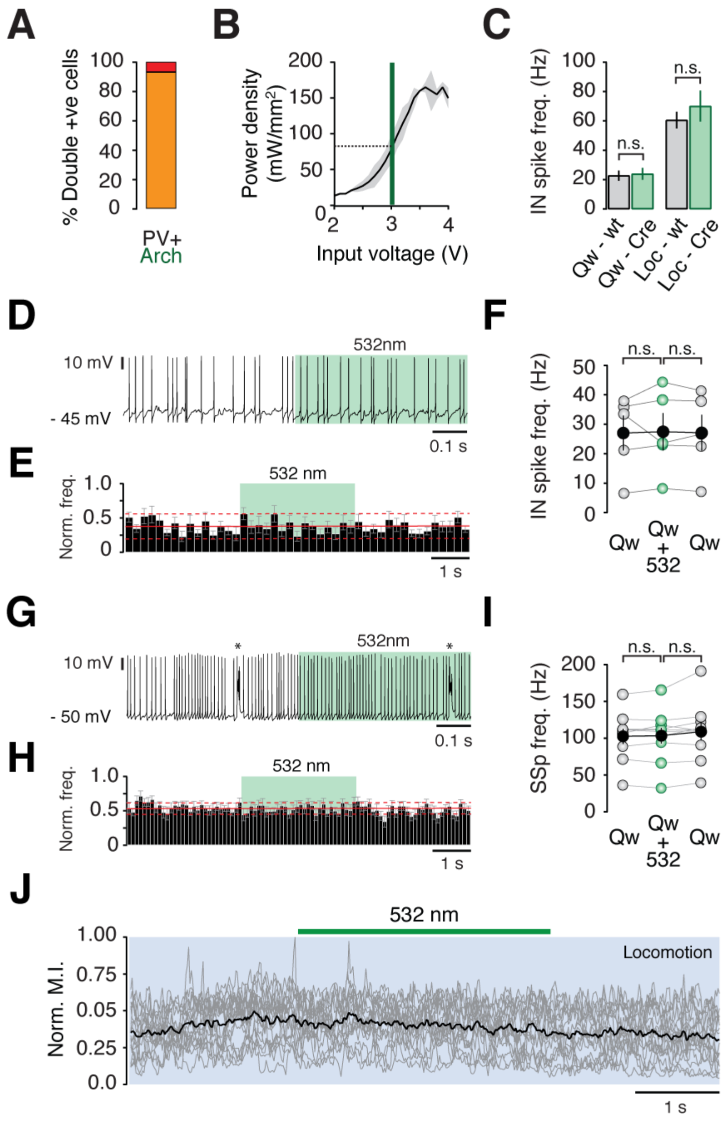

Supplementary Fig. 8. Effects of light stimulation in the absence of Arch 3.0 expression.

(**A**) Percentage of parvalbumin immunopositive molecular layer interneurons (red) that express Arch 3.0 (green) (93.1 ± 2.4 %, orange) (*n* = 16 slices from N = 4 mice).

(**B**) Laser power density (532 nm) as a function of increasing input voltage (V). Vertical green line represents the voltage used during optogentic stimulation, horizontal dashed line depicts the resulting power density (~ 70 mW/mm^2).

(**C**) Average molecular layer interneuron firing frequency during quiet wakefulness (Qw) and locomotion (Loc) in wild type (wt) and *Nos1Cre* (Cre) mice. ns = not significant, two-tailed *t* test.

(**D**) Representative voltage trace from a molecular layer interneuron in a *Nos1Cre* mouse in the absence of Arch 3.0 expression during quiet wakefulness. Green shading indicates the duration of 532 nm light stimulation.

(**E**) Molecular layer interneuron spike frequency histogram (bin size = 200 ms) from *Nos1Cre* mice in the absence of Arch 3.0 expression during quiet wakefulness (*n* = 5 from N = 4 mice). Green shading indicates the duration of 532 nm light stimulation. Solid red line indicates the average frequency before light stimulation, dashed lines indicate 2 x standard deviation of the mean.

(**F**) Average firing frequency of molecular layer interneurons in the absence of Arch 3.0 expression during quiet wakefulness before (Qw), during (Qw + 532) and after (Qw) 532 nm light stimulation. Grey symbols and connecting lines represent data from individual interneurons, black symbols represent mean ± s.e.m., ns = not significant, two-tailed *t* test (*n* = 5 cells from N = 4 mice).

(**G**) Representative voltage trace from a Purkinje cell in a *Nos1Cre* mouse in the absence of Arch 3.0 expression during quiet wakefulness. Green shading indicates the duration of 532 nm light stimulation, asterisks indicate complex spikes.

(**H**) Purkinje cell spike frequency histogram (bin size = 100 ms) from *Nos1Cre* mice in the absence of Arch 3.0 expression during quiet wakefulness (*n* = 10 from N = 7 mice). Green shading indicates the duration of 532 nm light stimulation. Solid red line indicates the average frequency before light stimulation, dashed lines indicate 2 x standard deviation of the mean.

(**I**) Average Purkinje cell SSp frequency in the absence of Arch 3.0 expression during quiet wakefulness before (Qw), during (Qw + 532) and after (Qw) 532 light stimulation. Grey symbols and connecting lines represent data from individual Purkinje cells, black symbols represent mean ± s.e.m., ns = not significant, two-tailed *t* test (*n* = 10 cells from N = 7 mice).

(**J**) The effects of 532 nm light stimulation (green bar, 3 sec) on normalized motion index (Norm M.I.) in *Nos1Cre* mice during locomotion (blue shading) in the absence of Arch 3.0 expression. Grey traces represent normalized motion index from individual cells, thick black line represents the average (*n* = 18 mice).

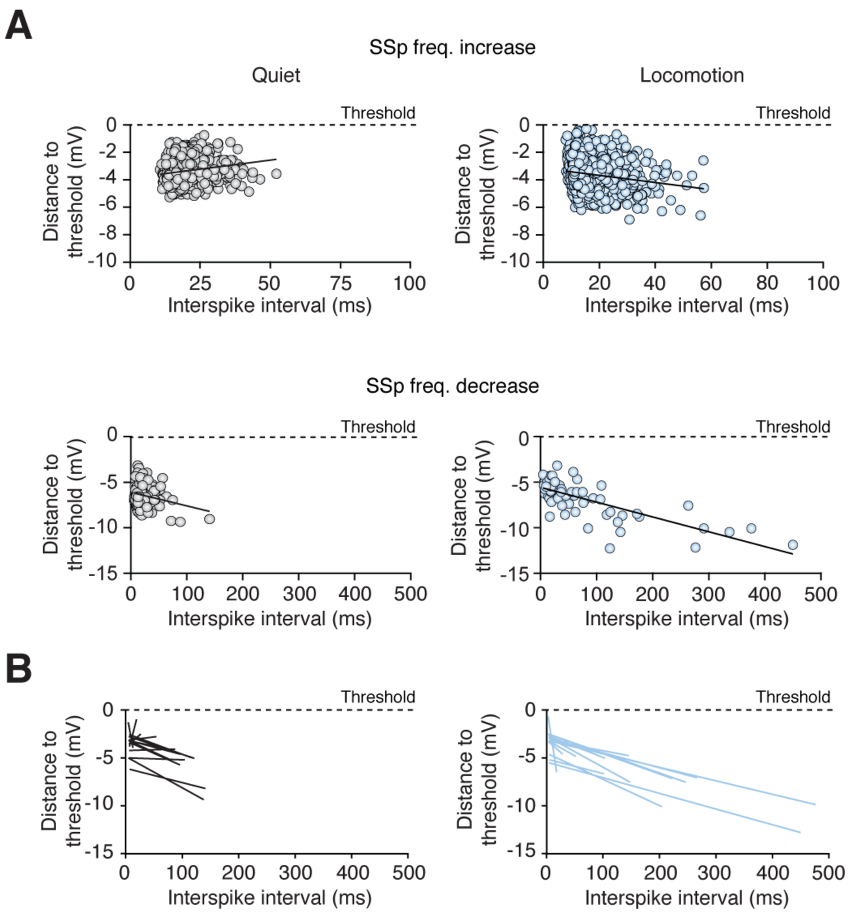

Supplementary Fig. 9. Changes in PC interspike intervals during locomotion.

(**A**) Relationship between SSp interspike modal V_m (expressed as distance to threshold) and interspike interval during quiet wakefulness (left) and locomotion (right) in two representative Purkinje cells. Filled circles represent individual ISIs, solid black line represents a linear fit to the data and dashed lines denote the average spike threshold. Note the increase in long duration interspike intervals in Purkinje cells that increase (upper panels) or decrease (lower panels) SSp frequency during locomotion.

(**B**) Relationship between SSp interspike modal V_m (expressed as distance to threshold) and interspike interval during quiet wakefulness (left) and locomotion (right) across a population of 14 Purkinje cells from N = 14 mice. Each solid line represents a linear fit to the distance to threshold vs interspike interval distribution in each cell. Dashed line represents the average spike threshold across Purkinje cells.

Purkinje cell electrophysiological properties	Mean ± s.d.	Range (min-max)	n
Resting membrane potential (mV)	-50.53 ± 2.28	-45.79 - (-54.05)	24
V_m standard deviation (mV)	1.53 ± 0.58	0.46 - 3.35	24
Spike threshold (mV)	-46.58 ± 2.77	-39.75 - (-50.44)	24
Firing rate (Hz)	66.90 ± 30.79	12.38 - 171.90	38
SSp interspike interval (ms)	18.71 ± 12.13	5.74 - 68.07	38
SSp ISI coefficent of variation	0.69 ± 0.48	0.13 - 2.92	38

Supplementary Table 1. Electrophysiological properties of PCs during quiet wakefulness.

Complex spike properties	Frequency (Hz)		ISI (ms)		CV_{ISI}		n
Somatic rec. - Qw	1.36 ± 0.28	ns	726.77 ± 139.38	ns	0.71 ± 0.14	ns	38
Dendritic rec. - Qw	1.46 ± 0.38		699.50 ± 191.11		0.72 ± 0.17		19

Supplementary Table 2. Complex spike and dendritic calcium spike firing properties during quiet wakefulness (mean ± s.d., ns = not significant).

Granule cell electrophysiological properties	Mean ± s.d.	Range (min-max)	n
Input resistance (MΩ)	438.99 ± 155.20	225.75 - 697.00	13
Capacitance (pF)	3.72 ± 0.77	2.05 - 4.86	13
Resting membrane potential (mV)	- 64.48 ± 5.41	- 73.66 - (-55.19)	13
V_m standard deviation (mV)	3.27 ± 1.20	0.97 - 5.39	13
Spike threshold (mV)	- 35.96 ± 5.18	- 40.28 - (- 26.79)	13
Firing rate (Hz)	0.14 ± 0.27	0 - 0.89	13

Supplementary Table 3. Electrophysiological properties of GCs during quiet wakefulness.

Granule cell spike burst statistics	Mean ± s.d.	Range (min-max)	n
Number of burst / s	0.74 ± 0.88	0.11 - 3.31	13
Intraburst frequency (Hz)	94.32 ± 25.74	59.84 - 141.80	13
Modal No. spikes / burst	3	3 - 168	13
Burst duration (ms)	76.14 ± 33.50	33.14 - 144.10	13
Intraburst CV_{ISI}	0.59 ± 0.14	0.40 - 0.85	13
Interburst CV_{ISI}	1.18 ± 0.38	0.55 - 1.86	13
1st spike latency (ms)	1006 ± 1046	88.20 - 3570	13

Supplementary Table 4. Granule cell burst statistics during locomotion.

Interneuron electrophysiological properties	Mean ± s.d.	Range (min-max)	n
Input resistance (MΩ)	250.81 ± 107.74	123.23 - 482.00	11
Capacitance (pF)	20.14 ± 7.43	9.40 - 33.60	11
Resting membrane potential (mV)	-47.40 ± 3.20	-52.87 - (-42.01)	13
V_m standard deviation (mV)	2.52 ± 0.71	1.34 - 3.57	13
Spike threshold (mV)	-40.92 ± 2.75	-45.59 - (-35.82)	13
Firing rate (Hz)	22.71 ± 16.47	4.20 - 58.09	23

Supplementary Table 5. Electrophysiological properties of MLIs during quiet wakefulness.

Dendritic calcium spikes	Frequency (Hz)	ISI (ms)	CV_{ISI}	n
Qw	1.56 ± 0.43	700.87 ± 232.08 ⌉ **	0.63 ± 0.08	6
Qw + Arch	2.10 ± 0.71	434.41 ± 112.99 ⌋	0.86 ± 0.23	6
Loc	1.46± 0.38 ⌉ **	779.26 ± 225.66 ⌉ **	0.77± 0.24	7
Loc + Arch	2.52 ± 0.58 ⌋	394.81 ± 79.24 ⌋	0.70 ± 0.22	7

Supplementary Table 6. PC dendritic calcium spike firing properties in the absence and presence of Arch 3.0 stimulation (*P* < 0.01, two-tailed *t* test).**

Purkinje cell complex spikes	Frequency (Hz)		ISI (ms)		CV_{ISI}	n
Qw	1.39 ± 0.18	**	706.23 ± 159.60	**	0.75 ± 0.26	16
Qw + Arch	2.86 ± 1.00		341.56 ± 116.10		0.71 ± 0.22	16
Loc	1.75 ± 0.63	*	570.97 ± 223.94	**	0.80 ± 0.28	16
Loc + Arch	4.06 ± 2.58		286.23 ± 113.31		0.75 ± 0.32	16

Supplementary Table 7. PC complex spike firing properties in the absence and presence of Arch 3.0 (*$P < 0.05$, **$P < 0.01$, two-tailed *t* test).
